# Switching Adsorbent Layered Material that Enables Stepwise Capture of C_8_ Aromatics via Single-Crystal-to-Single-Crystal Transformations

**DOI:** 10.1021/acs.chemmater.3c01920

**Published:** 2023-11-30

**Authors:** Mei-Yan Gao, Shi-Qiang Wang, Andrey A. Bezrukov, Shaza Darwish, Bai-Qiao Song, Chenghua Deng, Catiúcia R. M. O. Matos, Lunjie Liu, Boya Tang, Shan Dai, Sihai Yang, Michael J. Zaworotko

**Affiliations:** † Department of Chemical Sciences, Bernal Institute, University of Limerick, Limerick V94 T9PX, Republic of Ireland; ‡ Department of Chemistry, University of Manchester, Manchester M13 9PL, United Kingdom; § Agency for Science, Technology and Research (A*STAR), Institute of Materials Research and Engineering (IMRE), 2 Fusionopolis Way, 138634 Republic of Singapore; ∥ College of Chemistry and Molecular Engineering, Beijing National Laboratory for Molecular Sciences, Peking University, Beijing 100871, China; ⊥ Department of Materials Science and Engineering, Southern University of Science and Technology, Shenzhen, Guangdong 518055, China

## Abstract

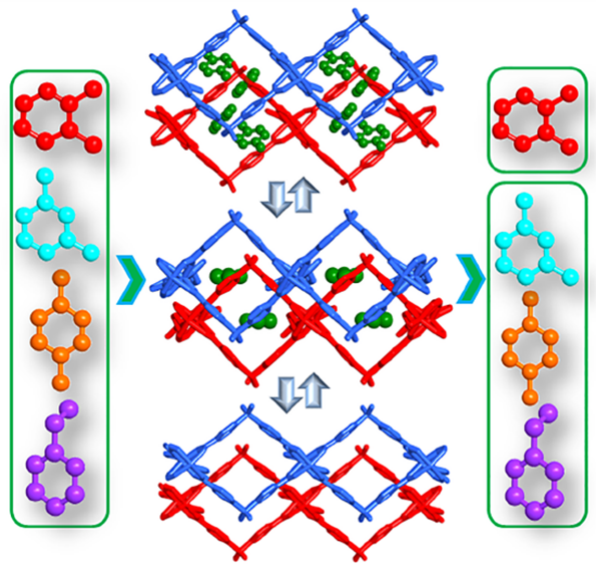

Separation of the C_8_ aromatic isomers, xylenes (PX, MX, and OX) and ethylbenzene (EB), is important to the petrochemical industry. Whereas physisorptive separation is an energy-efficient alternative to current processes, such as distillation, physisorbents do not generally exhibit strong C_8_ selectivity. Herein, we report the mixed-linker square lattice (**sql**) coordination network [Zn_2_(sba)_2_(bis)]_*n*_·*m*DMF (**sql-4,5-Zn**, H_2_sba or **4** = 4,4′-sulfonyldibenzoic acid, bis or **5** = trans-4,4′-bis(1-imidazolyl)stilbene) and its C_8_ sorption properties. **sql-4,5-Zn** was found to exhibit high uptake capacity for liquid C_8_ aromatics (∼20.2 wt %), and to the best of our knowledge, it is the first sorbent to exhibit selectivity for PX, EB, and MX over OX for binary, ternary, and quaternary mixtures from gas chromatography. Single-crystal structures of narrow-pore, intermediate-pore, and large-pore phases provided insight into the phase transformations, which were enabled by flexibility of the linker ligands and changes in the square grid geometry and interlayer distances. This work adds to the library of two-dimensional coordination networks that exhibit high uptake, thanks to clay-like expansion, and strong selectivity, thanks to shape-selective binding sites, for C_8_ isomers.

Separation of the C_8_ aromatic isomers *ortho*-xylene (OX), *meta*-xylene (MX), *para*-xylene (PX), and ethylbenzene (EB) is regarded as one of the seven industrially significant separations “to change the world”.^1^ Each of the pure C_8_ isomers is an important chemical commodity:^2−4^ OX is the precursor to phthalic anhydride for coatings and plasticizers; MX is used for isophthalic acid and isophthalic nitrite; PX has utility for the manufacture of polybutylene terephthalate (PBT) and polyethylene terephthalate (PET); EB is used as a petrochemical intermediate to produce the resin monomer styrene. EB also plays a role in the pharmaceutical industry to produce the drug substances synthomycin and chloramphenicol. However, the separation of C_8_ aromatics is challenging, as OX, MX, PX, and EB exhibit similar physicochemical properties, including molecular sizes and boiling points (Table S1).^5−7^ As a result, conventional distillation processes are energy-intensive.^8,9^ At present, the most widely used industrial technique for separation of C_8_ aromatics uses zeolites in simulated moving beds under a high temperature (180 °C) and pressure (9 bar).^10,11^

In this context, physisorbent-based separation technologies represent an attractive alternative to existing C_8_ purification methods.^2,7,12−14^ However, widespread implementation of physisorbents is hindered by low capacity and selectivity; *e.g*., FAU zeolites exhibit limited selectivity (∼5) and working capacity (∼10%).^15^ There is therefore a need to investigate new classes of physisorbents^6,7^ as exemplified by flexible molecular compounds, which exhibit high adsorptive separation of *m*-xylene, although with low uptake.^16−18^

Metal–organic materials (MOMs),^19−21^ which include porous coordination polymers (PCPs)^22^ and metal–organic frameworks (MOFs),^23^ offer potential utility in storage, separation, sensing, and catalysis.^24−29^ An advantage of MOMs is that they can offer structural tunability in terms of pore size, shape, and chemistry to enable systematic crystal engineering studies of their structure/property relationships.^2,3,7,12,30,31^ Of these, “0D materials”, such as Werner complexes and L-shaped Ag(I) molecular complexes, can exhibit OX or PX adsorption preference, but they lack the combination of high selectivity (>5) and high working capacity.^32,33^ Farha et al. demonstrated two examples of MOFs, **NU-2000** and **NU-2001**, that exhibit strong separation performance of xylene isomers in 2022.^8^ In the same year, a flexible stacked coordination polymer, [Mn(dhbq)(H_2_O)_2_] (H_2_dhbq = 2,5-dihydroxy-1,4-benzoquinone), was reported by Li and co-workers that exhibits recognition and sieving of xylene isomers.^2^ Recently, we reported two switching adsorbent layered materials (SALMAs) **sql-1-Co-NCS** (single-linker, bipy) and **sql-1,3-Co-NCS** (mixed-linker, bipy, bptz), which exhibit switching behavior triggered by different C_8_ aromatics at different switching pressures along with high selectivity.^4,34^ Switching or flexible sorbents can efficiently separate sorbate mixtures thanks to selective binding and fast diffusion.^35,36^ However, despite growing interest in switching sorbents, their utility for the separation of C_8_ aromatics remains understudied. Additionally, disorder of guest molecules can hinder the use of SCXRD to gain insight into C_8_ aromatic-loaded phases.^4^ Indeed, to our knowledge, there has been no report of a sorbent that exhibits stepwise loading (Scheme 1) of C_8_ isomers for which the SCXRD structures of both fully and partially C_8_-loaded phases have been determined.

**Scheme 1:**
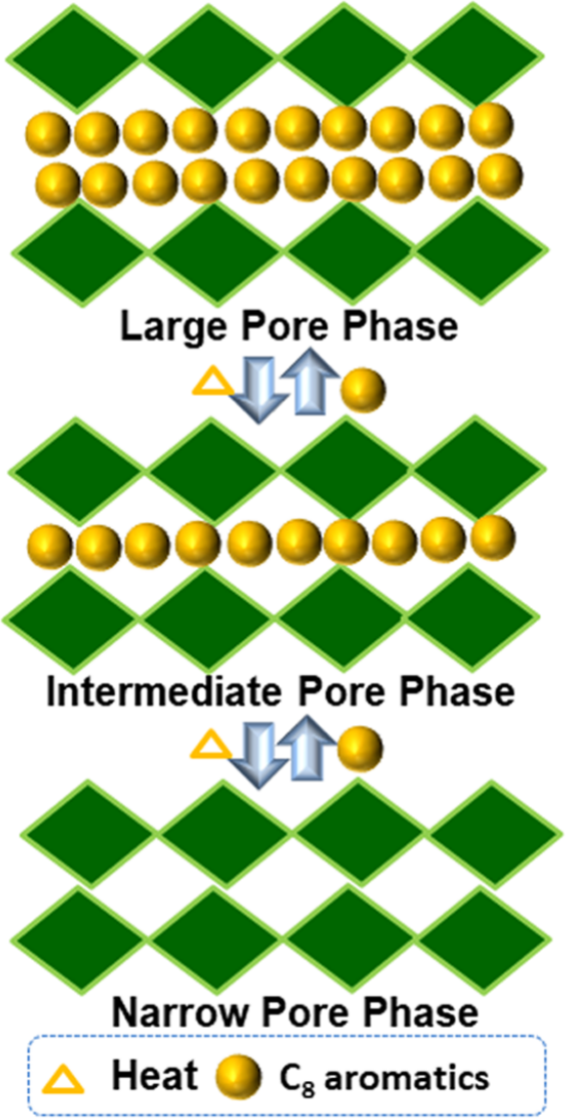
Schematic Illustration of Stepwise Sorption by a Layered Sorbent

Herein, we report such a material, the square lattice, **sql**, topology coordination network composed of the mixed-linker ligands 4,4′-sulfonyldibenzoic acid (sba) and trans-4,4′-bis(1-imidazolyl)stilbene (bis), [Zn_2_(sba)_2_(bis)]_*n*_·*m*DMF (**sql-4,5-Zn**). This is the first report of the use of bis in a MOF. **sql-4,5-Zn** was characterized through SCXRD, thermogravimetric analysis (TGA), powder X-ray diffraction (PXRD), and variable-temperature PXRD (VT-PXRD). The gas/vapor sorption and C_8_ aromatic separation properties of **sql-4,5-Zn** are also presented.

## Experimental Section

All reagents and solvents were commercially available and were used without further purification. *trans*-4,4′-Bis(1-imidazolyl)stilbene was made following a previously published method.^37^^1^H NMR (400 MHz, DMSO-*d*_6_): δ = 7.45 (s, 1H), 7.78 (s, 3H), 7.87 (d, 2H, *J* = 8 Hz), 8.14 (s, 1H), 9.55 (s, 1H) (Figure S18).

### Synthesis of [Zn_2_(sba)_2_(bis)]_*n*_·*m*DMF (**sql-4,5-Zn-α**)

A mixture of Zn(NO_3_)_2_·6H_2_O (60 mg, 0.2 mmol), 4,4′-sulfonyldibenzoic acid (10 mg, 0.032 mmol), *trans*-4,4′-bis(1-imidazolyl)stilbene (10 mg, 0.032 mmol), and DMF (3 mL) was added to a 20 mL glass vial and then subjected to ultrasonication for 30 s. The vial was capped and placed in an oven at 105 °C. After 24 h, the vial was removed from the oven and allowed to cool to room temperature. Colorless block crystals were harvested by filtration and washed with DMF (yield: 50%).

[Zn_2_(sba)_2_(bis)]_*n*_ (**sql-4,5-Zn-β**) was obtained by activating the acetonitrile-exchanged (MeCN) phase of **sql-4,5-Zn-α** under vacuum overnight.

[Zn_2_(sba)_2_(bis)·2OX]_*n*_ (**sql-4,5-Zn·2OX**), [Zn_2_(sba)_2_(bis)·2MX]_*n*_ (**sql-4,5-Zn·2MX**), [Zn_2_(sba)_2_(bis)·2PX]_*n*_ (**sql-4,5-Zn·2PX**), and [Zn_2_(sba)_2_(bis)·2EB]_*n*_ (**sql-4,5-Zn·2EB**) were obtained by soaking the MeCN-exchanged phase of **sql-4,5-Zn-α** in OX, MX, PX, and EB, respectively, for 3 h.

[Zn_2_(sba)_2_(bis)·MX]_*n*_ (**sql-4,5-Zn·1MX**) was obtained by heating the fully MX-loaded phase of **sql-4,5-Zn·2MX** at 80 °C for ∼15 min on the goniometer head of a single-crystal X-ray diffraction (SCXRD) instrument under nitrogen flow.

[Zn_2_(sba)_2_(bis)·PX]_*n*_ (**sql-4,5-Zn·1PX**) was obtained by heating the PX-loaded phase, **sql-4,5-Zn·2PX**, at 85 °C for ∼2 h.

[Zn_2_(sba)_2_(bis)·EB]_*n*_ (**sql-4,5-Zn·1EB**) was obtained by heating the EB-loaded phase, **sql-4,5-Zn·2EB**, at 85 °C for ∼2 h.

## Results

**Figure 1:**
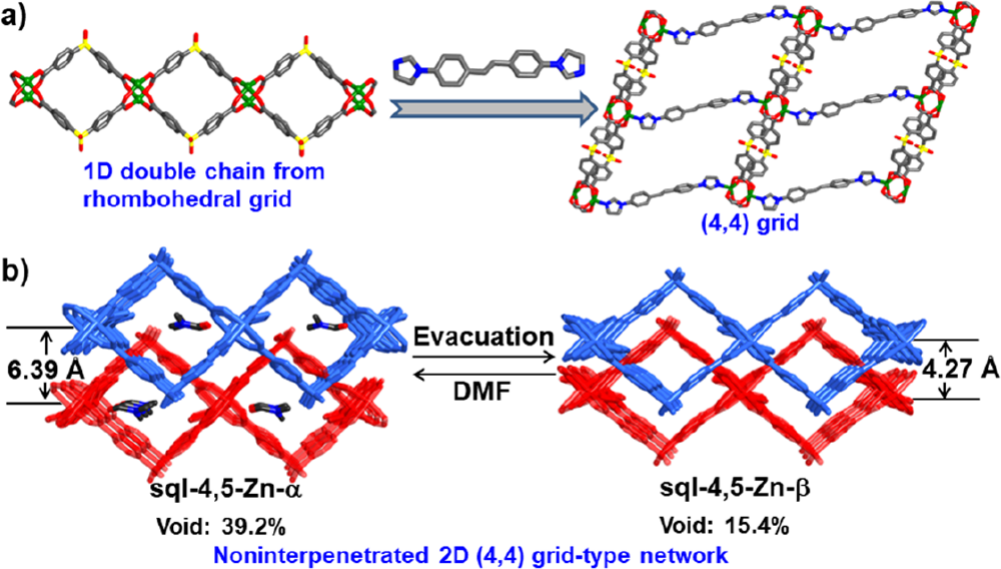
(a) **sql-4,5-Zn-α** is comprised of a 1D spiro chain linked by bis ligands to form a rhombic (4,4) grid; (b) reversible transformations between **sql-4,5-Zn-β** and **sql-4,5-Zn-α**. Atom color code: green, Zn; red, O; gray, C; blue, N; yellow S.

Single crystals of the as-synthesized large-pore (LP) phase, [Zn_2_(sba)_2_(bis)]_*n*_·*m*DMF (**sql-4,5-Zn-α**), were prepared through solvothermal synthesis at 105 °C. Activation of the acetonitrile-exchanged (MeCN) phase of **sql-4,5-Zn-α** under vacuum overnight at room temperature afforded single crystals of the narrow-pore (NP) phase, **sql-4,5-Zn-β** (Figure 1). The LP phase of **sql-4,5-Zn-α** can also be transformed to **sql-4,5-Zn-β** if single crystals are left in air for 5 min. The phase purities of **sql-4,5-Zn-α** and **sql-4,5-Zn-β** were supported by their powder X-ray diffraction (PXRD) patterns (Figure S1). PXRD patterns of the β phase after soaking in DMF indicate that the α–β transformation is reversible (Figure S1). **sql-4,5-Zn-β** retained crystallinity to >350 °C as revealed by VT-PXRD (starting from α) and TGA data (Figures S4 and S5). Additionally, the single crystals of **sql-4,5-Zn** still present good diffraction images after soaking in various solvents for more than 24 h, indicating its excellent stability in these solvents (Figure S27).

SCXRD studies revealed that **sql-4,5-Zn-α** and **sql-4,5-Zn-β** crystallized in the triclinic space group *P*-1 (Table S3). As shown in Figure S7, the crystal structure of **sql-4,5-Zn-α** is based upon sba linkers that coordinate Zn^2+^ to afford paddle-wheel units. The sba linkers are bent at 99.351°/99.428°, and they form spiro chains of paddle wheels (Figure 1a). These chains are connected axially with bis ligands to afford a (4,4) grid with an **sql** topology and no interpenetration (Figure 1b). **sql-4,5-Zn-α** is the first crystal structure of an MOF that exploits bis as a linker ligand (Figure S8). The distances between adjacent chains and layers in **sql-4,5-Zn-α** are 20.4847 and 6.3893 Å, respectively (Table 1). The void volumes of the unit cells for LP and NP phases were calculated using PLATON to be 39.2 and 15.4%, respectively (Table S6).^38^ When DMF molecules were removed, the guest free NP phase **sql-4,5-Zn-β** was obtained, with distances between adjacent layers and chains of 4.2670 and 21.1282(1) Å, respectively. The angle subtended by sba ligands is 105.229(9)° (Figures S9 and S10 and Table 1).

**Table 1:**
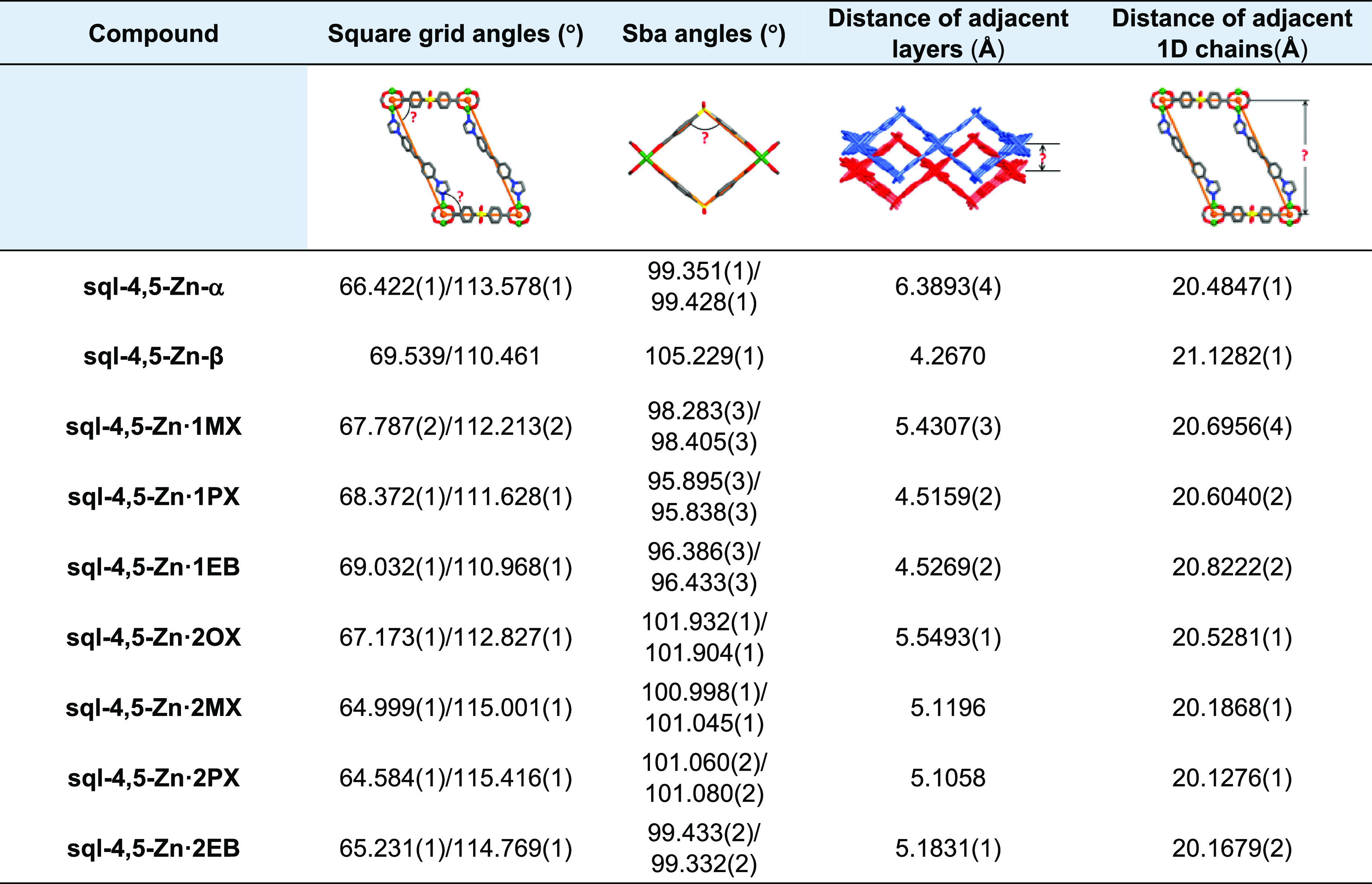
Structural Parameters of **sql-4,5-Zn-α** and **sql-4,5-Zn-β** and Their Fully and Partially C_8_ Aromatic-Loaded Phases

**Figure 2:**
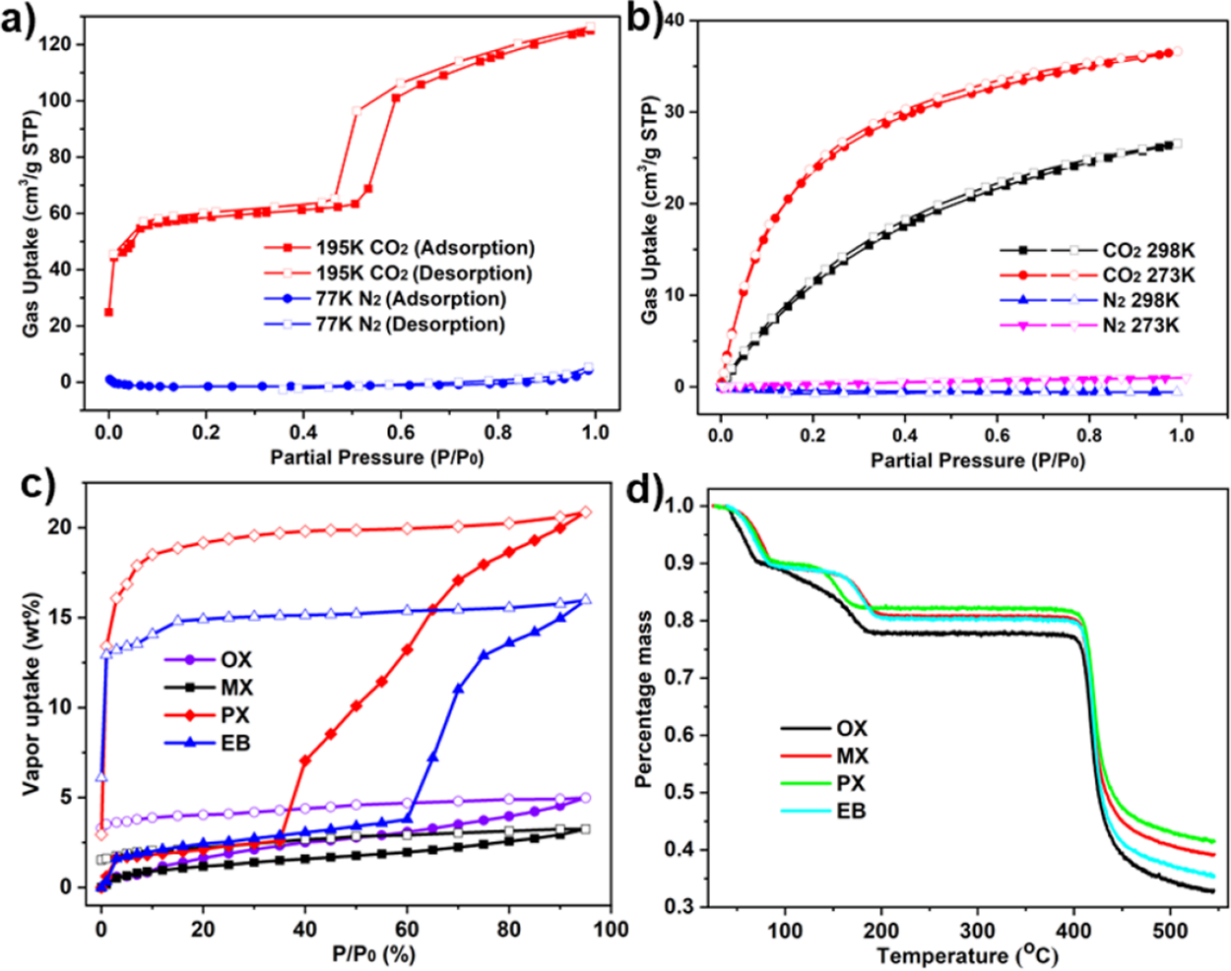
(a) CO_2_ and N_2_ isotherms for **sql-4,5-Zn-β** recorded at 195 and 77 K. (b) CO_2_ and N_2_ isotherms for **sql-4,5-Zn-β** recorded at 273 and 298 K. (c) C_8_ aromatic vapor sorption isotherms for **sql-4,5-Zn-β** recorded at 298 K. Solid and open symbols represent adsorption and desorption, respectively. (d) TGA profiles for the solvent soaking experiments after liquid soaking in xylenes and ethylbenzene.

To study the porosity of **sql-4,5-Zn**, CO_2_ and N_2_ gas sorption isotherms at low temperatures were measured (Figure 2a). Prior to conducting gas sorption tests, **sql-4,5-Zn-β** was treated for 12 h under dynamic vacuum at room temperature. For CO_2_ adsorption at 195 K, a two-step sorption isotherm consistent with a narrow-pore to wide-pore gas-induced transformation was observed (type F–II).^39^ The first plateau showed an uptake of 62 cm^3^ g^–1^ at ∼380 mmHg (three CO_2_ molecules per formula unit), at which point a step occurred that resulted in a saturated CO_2_ uptake of 121 cm^3^ g^–1^ at 760 mmHg (six CO_2_ molecules per formula unit). This two-step isotherm is consistent with a CO_2_-induced phase transformation. The Langmuir surface areas for **sql-4,5-Zn-β** and **sql-4,5-Zn-α** in the first and second steps calculated from the CO_2_ sorption isotherm at 195 K are 289 and 870 m^2^ g^–1^, respectively (Figures S25 and S26). The CO_2_ sorption profile was found to be consistent over three consecutive cycles, indicating the recyclability of **sql-4,5-Zn** (Figure S17). In the case of N_2_ sorption at 77 K, no appreciable sorption was observed, indicating that **sql-4,5-Zn-β** is nonporous to N_2_. The two-step CO_2_ sorption and lack of N_2_ sorption are similar to **Cu(bpp)**_**2**_**(BF**_**4**_**)**_**2**_ and **sql-1,3-Co-NCS**.^40,41^**sql-4,5-Zn-β** also served as a sieve for CO_2_ over N_2_ at 273 and 298 K (Figure 2b). These observations are consistent with the small pore window of the β phase (2.18 Å) and with transient porosity^42^ enabling sorption of CO_2_ molecules.

Dynamic vapor sorption (DVS) studies of C_8_ for **sql-4,5-Zn-β** were conducted at 298 K, resulting in type F–I isotherms for PX and EB, but only surface sorption was observed for OX and MX (Figure 2c). The uptakes of PX and EB for **sql-4,5-Zn-β** were measured to be around 20.7 and 15.9 wt %, respectively. On desorption, the PX and EB isotherms revealed large hysteresis, which is very common for gate-opening materials.^4,21,34^

Single crystals of the C_8_-loaded LP phases were obtained by soaking the MeCN-exchanged phase of **sql-4,5-Zn-α** in the corresponding C_8_ aromatics: **sql-4,5-Zn·2OX**, **sql-4,5-Zn·2MX**, **sql-4,5-Zn·2PX**, and **sql-4,5-Zn·2EB**. SCXRD studies revealed that all four phases crystallized in the triclinic space group *P*-1 (Table S4). There are two independent C_8_ molecules per formula unit, one located between layers and the other in cavities. The distance between adjacent layers lies between those of **sql-4,5-Zn-α** and **sql-4,5-Zn-β** (Table 1). Additionally, the distances between 1D chains, the bend angles in the sba ligands, the dihedral angles of the aromatic rings in the N donor ligand, and paddle-wheel units varied depending upon the guest (Table 1, Table S7, and Figures S11–S15). The phase purities of the C_8_-loaded phases were supported by the PXRD data (Figure S2). TGA profiles of the C_8_-loaded phases indicate that C_8_ molecules were removed stepwise at *ca*. 85 and *ca*. 150 °C to afford **sql-4,5-Zn-β**, which remained until 400 °C (Figure 2d). Weight losses were 9.2 and 11.8 wt % for the OX-loaded phase, 9.9 and 8.7 wt % for the MX-loaded phase, 9.4 and 8.0 wt % for the PX-loaded phase, and 9.1 and 8.6 wt % for the EB-loaded phase. The calculated weight loss from SCXRD is 8.4 wt % for each mole of guest.

**Figure 3:**
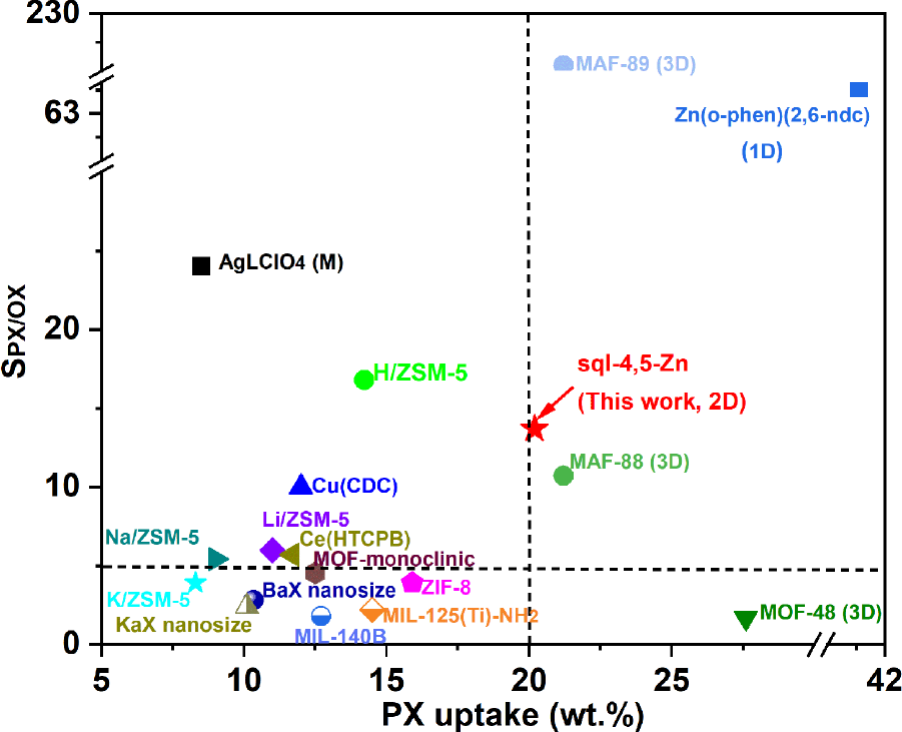
Comparison of physisorbents in terms of their PX adsorption capacity and PX/OX selectivity.

The different gate-opening pressures of **sql-4,5-Zn-β** in response to C_8_ aromatics are indicative of potential utility for physisorptive separation. Therefore, vapor/liquid binary mixture separation tests were performed on **sql-4,5-Zn-β**, and selectivities were determined using ^1^H NMR and gas chromatography (GC) (Figures S19–S24 and S28–S32 and Tables S14–S16). It is noteworthy that **sql-4,5-Zn-β** exhibited selectivity from both equimolar binary liquid (*S*_PX/OX_ = 13.69, *S*_EB/OX_ = 16.92, and *S*_MX/OX_ = 4.23 from GC and *S*_PX/OX_ = 8.0, *S*_EB/OX_ = 7.2, and *S*_MX/OX_ = 2.0 from ^1^H NMR) and equimolar binary vapor phases (*S*_PX/OX_ = 16.56, *S*_EB/OX_ = 13.65, and *S*_MX/OX_ = 3.43 from GC) (Tables S9 and S14–S16). It was also selective for equimolar ternary and quaternary liquid and vapor phases from GC. The ratios of PX/MX/OX, EB/PX/OX, EB/MX/OX, and PX/EB/MX/OX for equimolar liquid mixtures are 19.01:3.08:1, 16.23:16.12:1, 12.49:3.65:1, and 18.53:13.67:3.96:1, respectively. Whereas **sql-4,5-Zn-β** is less selective for PX/OX than **H/ZSM-5**, its PX uptake is higher than most materials (Figure 3).^43^ To the best of our knowledge, there are reports on highly selective separation of PX/OX and MX/OX, whereas highly selective separation of EB/OX is less reported. Although the rigid sorbents **MAF-89** exhibit higher PX uptake and PX/OX selectivity, the mechanism for **sql-4,5-Zn** involves flexibility like the dynamic framework **Zn(o-phen)(2,6-ndc)**, which was termed a “dynamic chemical clip”.^36,44^ Switching or flexible materials for C_8_ separation are uncommon, especially for layered structures (Table S10). Additionally, when compared with the selective materials for the OX listed in Table S11, inverse-selectivity materials such as that herein can in effect directly capture a target xylene from the OX, simplifying the separation process.

## Discussion

According to the literature, **sql** nets account for *ca*. 45% of 2D coordination networks.^45^ Our survey of the TOPOS TTO**∩**CSD^46^ revealed that there are single-linker and mixed-linker **sql** nets, with **sql-1-Co-NCS** and **sql-1,3-Co-NCS** being representative of these two classes, respectively.^4,34^ While mixed-linker **sql** nets based on dicarboxylate linkers and ditopic N donor ligands are common, there are only 47 examples of paddle-wheel and coplanar layers of **sql** nets with “V-shaped” dicarboxylate linkers (Table S2). These materials were studied for magnetism, luminescence, dye degradation, and other applications but until now were not studied with respect to C_8_ aromatics separation.

**Figure 4:**
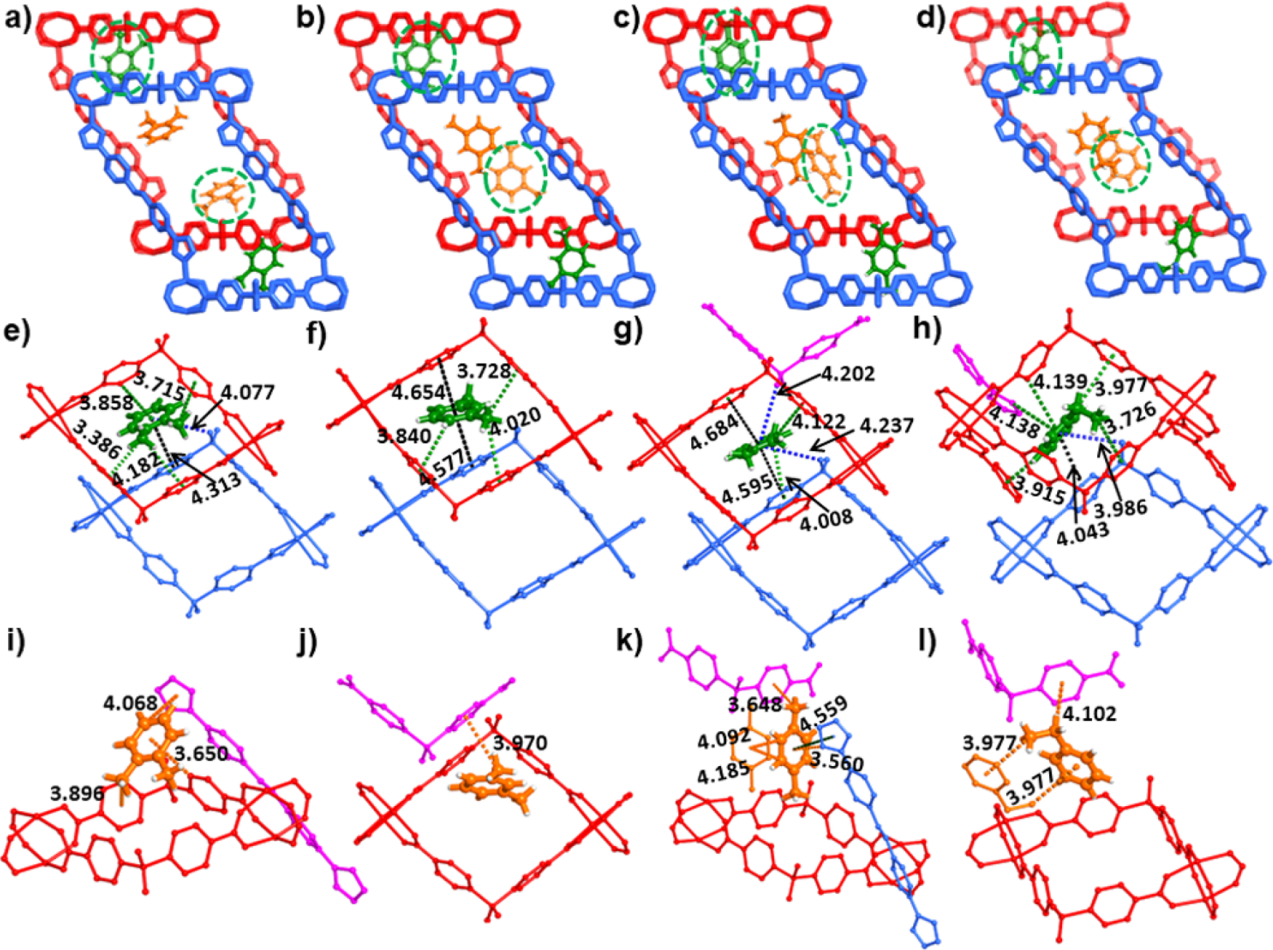
Structures of four C_8_ aromatic-loaded phases of **sql-4,5-Zn**. (a–d) Layering of fully guest-loaded phases for **sql-4,5-Zn·2OX**, **sql-4,5-Zn·2MX**, **sql-4,5-Zn·2PX**, and **sql-4,5-Zn·2EB** (green, C_8_ aromatics exist in the square cavities; orange, C_8_ aromatics in the interlayer/intralayer spaces). (e–h) Host–guest interactions in the square cavities (marked with a circle in the above arrays) of the OX-, MX-, PX-, and EB-loaded phases. (i–l) Host–guest interactions in the interlayer spaces (marked with a circle in the above arrays) of the OX-, MX-, PX-, and EB-loaded phases. The π···π interactions between guest molecules and the framework are highlighted in black dashed lines; the C–H···π interactions between guest molecules and the framework are highlighted in green or black dashed lines; the π···O interactions between guest molecules and the framework are highlighted in blue dashed lines. The closest contacts between the framework and adsorbates are highlighted (Å). The color of the framework from front to back was light blue, red, and pink.

In order to understand the mechanism for selectivity and capacity for C_8_ aromatics, the crystal structures of the C_8_ aromatic-loaded phases were determined by SCXRD. As shown in Figure 4a–d, C_8_ aromatic guests lie within the square cavities (green) and between the layers (orange), comprising ∼37.7, ∼39.1, ∼39.3, and 39.4% of the unit-cell volume in **sql-4,5-Zn·2OX**, **sql-4,5-Zn·2MX**, **sql-4,5-Zn·2PX**, and **sql-4,5-Zn·2EB**, respectively (Table S6). There are two C_8_ aromatic molecules per formula unit (1.9 mmol/g or 20.2 wt %). The uptake of PX from soaking is consistent with the saturation of PX uptake from vapor sorption.

Although there are the same numbers of guest molecules per formula unit in all four C_8_ aromatic-loaded phases, some differences in their host–guest interactions were found (Figure 4a–d). For the guests in the square cavities (Figure 4–h and Table S13), C_8_ aromatics exhibit C–H···π and π···π stacking interactions with the benzene and/or imidazole moieties, as well as O···π interactions with the oba ligand. PX and EB showed host–guest interactions with the nearest three layers (blue, red, and pink in Figure 4), while MX and OX exhibited only host–guest interactions with two layers (blue and red in Figure 4). With respect to the guests located in the interlayer/intralayer spaces (Figures 4i–l and Table S13), only π···π stacking interactions were observed between PX and imidazole rings. In addition, there are C–H···π interactions with other guests for PX and EB, while OX and MX present only C–H···π interactions with the host framework. These different host–guest/guest–guest interactions can be ascribed to the different shapes of the C_8_ molecules, which ultimately leads to the high selectivity for PX and EB over OX. The presence of two xylene molecules per formula unit resulted in interlayer separations increasing from 4.2670 (NP phase) to >5.1058 Å (LP or C_8_-loaded phases, Table 1). This clay-like expansion is similar to that reported for **sql-1-Co-NCS**.^34^ Additionally, the horizontal shift of AA layers (3 in Figure S16) increased from 11.7719 to >14.2710 Å (Table S8).

**Figure 5:**
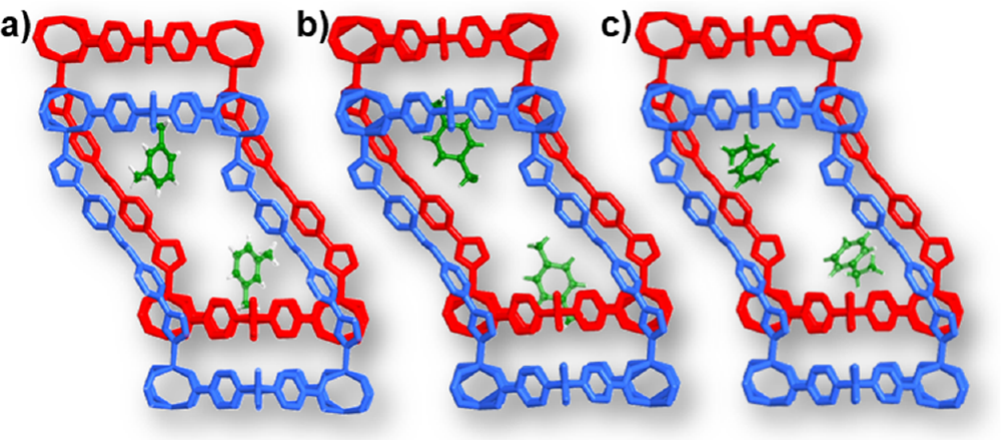
Structures of partially guest-loaded phases of (a) **sql-4,5-Zn·1MX**, (b) **sql-4,5-Zn·1PX**, and (c) **sql-4,5-Zn·1EB**.

The stepwise removal of C_8_ aromatics indicated by the TGA curves prompted us to isolate the partially guest-loaded phases (intermediate-pore, IP, phases) of **sql-4,5-Zn·1MX**, **sql-4,5-Zn·1PX**, and **sql-4,5-Zn·1EB** from the respective fully loaded LP phases. **sql-4,5-Zn·1MX**, **sql-4,5-Zn·1PX**, and **sql-4,5-Zn·1EB** were heated at 80 °C for about 15 min, 85 °C for about 2 h, and 85 °C for about 2 h, respectively. SCXRD studies revealed that the IP phases retained the space group *P*-1, and PXRD patterns indicated phase purity (Table S5 and Figure S3). Although there are two previous examples of stepwise removal of C_8_ aromatics, the single-crystal structures of the partially loaded phases were not reported.^9,34^ Therefore, we report herein the first SCXRD structures of the IP C_8_-loaded phases (Table S12). Further heating of **sql-4,5-Zn·1MX**, **sql-4,5-Zn·1PX**, and **sql-4,5-Zn·1EB** at 85 °C for about 2 h, 160 °C for 3 h, and 200 °C for 3 h, respectively, resulted in transformation to **sql-4,5-Zn-β**. The stepwise guest removal processes were followed by VT-PXRD (Figure S6). **sql-4,5-Zn-β** can also be obtained from **sql-4,5-Zn·2OX** by leaving the crystals in air overnight. The single-crystal structure of **sql-4,5-Zn·1OX** was not determined, as it lost crystallinity. These findings indicate that interactions between C_8_ aromatics and pore walls have the following hierarchy: EB and PX > MX > OX, consistent with the measured selectivities. As shown in Table S6, C_8_ aromatic guests in **sql-4,5-Zn·1MX**, **sql-4,5-Zn·1PX**, and **sql-4,5-Zn·1EB** occupy 25.2, 25.1, and 25.6% of the unit-cell volume, respectively, which are less than the fully guest-loaded phases (39.1% for **sql-4,5-Zn·2MX**, 38.3% for **sql-4,5-Zn·2PX**, and 38.4% for **sql-4,5-Zn·2EB**) and more than **sql-4,5-Zn-β** (15.4%) (Table S6). Additionally, sba angles, distances between adjacent layers, and 1D chains lie between those of the NP and LP phases (Table 1). When comparing the IP phases to the fully loaded structures, the C_8_ aromatics in the interlayer spaces had been removed, while those in the square cavities were retained in a slightly changed location (Figure 5). These results indicate stronger binding interactions in the square cavities than in the interlayer spaces, which is consistent with the aforementioned analysis of intermolecular interactions. Interestingly, our attempts to obtain the partially OX-loaded phase were unsuccessful, presumably due to weaker interactions between the OX and the framework.

## Conclusions

In summary, we report a layered coordination network, **sql-4,5-Zn**, the first example of a mixed-linker material with a “V-shaped” dicarboxylate ligand that exhibits selectivity for C_8_ aromatics. SCXRD analysis of the guest-loaded phases of **sql-4,5-Zn** reveals the expected clay-like contraction of layers from LP to IP to NP phases. We attribute these properties to the flexibility of the N donor and dicarboxylate ligands as well as the clay-like nature. **sql-4,5-Zn** is the first sorbent of anytype to exhibit high selectivity for both PX and EB over OX (>10, GC), competitive selectivity for MX over OX (3.0, GC) and high adsorption capacity for PX and EB (∼20.2 wt %). The modular nature of the mixed-linker **sql** framework enables it to serve as the prototype for a platform of switching adsorbent layered materials (SALMAs) to address C_8_ aromatic separation challenges.

## Supporting Information Available

Experimental and characterization details; additional figures and images; PXRD patterns; VT-PXRD patterns; TGA analysis, ^1^H NMR, GC results, and sorption isotherms (PDF)
Crystallographic data for **sql-4,5-Zn-α** (CIF)

## Supplementary Material

cm3c01920_si_001.pdf

cm3c01920_si_002.cif
