# Supplementary material for: Switching Adsorbent Layered Material that Enables Stepwise Capture of C8 Aromatics via Single-Crystal-to-Single-Crystal Transformations
Source: Chem Mater. 2023 Nov 30;35(23):10001–8. doi: 10.1021/acs.chemmater.3c01920 (PMC10720335; doi:10.1021/acs.chemmater.3c01920)
Supplement: Supplementary file 1 — cm3c01920_si_001.pdf [file cm3c01920_si_001.pdf]

Supplementary Information for

Switching adsorbent layered material that enables stepwise capture of C_8 aromatics via single-crystal-to-single-crystal transformations

Mei-Yan Gao,[a] Shi-Qiang Wang,[a,c] Andrey A. Bezrukov,[a] Shaza Darwish,[a] Bai-Qiao Song,[a] Chenghua Deng,[a] Catiúcia R. M. O. Matos,[a] Lunjie Liu,[e] Boya Tang,[b] Shan Dai,[b] Sihai Yang,[b,d] and Michael J. Zaworotko*,[a]

[a] Department of Chemical Sciences, Bernal Institute, University of Limerick, Limerick V94 T9PX, Republic of Ireland E-mail: xtal@ul.ie

[b] Department of Chemistry, University of Manchester, Manchester, M13 9PL, U.K.

[c] Institute of Materials Research and Engineering (IMRE), Agency for Science, Technology and Research (A*STAR), 2 Fusionopolis Way, Singapore 138634, Republic of Singapore

[d] College of Chemistry and Molecular Engineering, Beijing National Laboratory for Molecular Sciences, Peking University, Beijing 100871, China

[e] Department of Materials Science and Engineering, Southern University of Science and Technology, Shenzhen, Guangdong, 518055, China

Table of Contents

1. Characterization and Methods. 4
1.1 Single Crystal X-ray diffraction 4
1.2 Powder X-ray diffraction (PXRD). 4
1.3 Thermogravimetric analysis (TGA) 5
1.4 In-situ Variable Temperature Powder X-ray Diffraction (VT-PXRD) 5
1.5 Low Pressure Gas Adsorption Studies 6
1.6 Dynamic Vacuum Vapor Sorption 6
1.7 C_8 aromatics selectivity studies using Nuclear Magnetic Resonance (NMR) 7
1.8 C_8 aromatics selectivity studies using Gas Chromatography (GC) 8
1.9 Calculation of the Langmuir surface area 10
1.10 The Antoine equation 11
2. Comparison of C_8 aromatics. 13
3. CSD search results 13
4. Crystallographic Data 17
5. Void volume 20
6. Powder X-ray Diffraction 20
7. Thermogravimetric analysis (TGA) 22
8. VT-PXRD 22
9. Paddle-wheel unit 23
10. CSD Survey of N-donor ligand 24
11. Comparison of Bond length/Distances and Angle for Crystal Structures 24
12. Angles of the Rings in Ditopic N-donor Ligand 27
13. Horizontal shift of the layers 28
14. Sorption Isotherms 29
15. ^{1}H NMR Spectra. 29
16. Langmuir fit 33
17. Stability Studies 34
18. GC Spectra 34
19. Comparison of MOMs with separation of C_8 aromatics 37
20. Interactions between Framework and C_8 Aromatics. 39

21. Separation results from GC and ^{1}H NMR 40
22. Coefficients for Antoine equation. 41
23. References 42

1. Characterization and Methods.

1.1 Single Crystal X-ray diffraction

Single crystal X-ray diffraction data of **sql-4,5-Zn-α**, **sql-4,5-Zn·2OX**, **sql-4,5-Zn·2MX**, **sql-4,5-Zn·2PX**, **sql-4,5-Zn·2EB**, **sql-4,5-Zn·1MX**, **sql-4,5-Zn·1PX**, and **sql-4,5-Zn·1EB,** were collected on a Bruker Quest diffractometer equipped with a IμS microfocus X-ray source Mo K_α ($\lambda = 0.71073$ Å) and CMOS detector. The structural data of **sql-4,5-Zn-β** was collected on a Bruker Quest diffractometer equipped with a CMOS detector and IμS microfocus X-ray source Cu K_α ($\lambda = 1.54178$ Å). APEX4 was used for collecting, indexing, integrating and scaling the data.[1] Absorption corrections were performed by multi-scan method.[2] Space groups were determined using XPREP[3] as implemented in APEX4. All the scaled data were solved using intrinsic phasing method (XT)[4] and refined on F^2 using SHELXL[5] inbuilt in OLEX2 v1.5 (2020) program.[6] All non-hydrogen atoms present in the frameworks were refined anisotropically. Hydrogen atoms were located at idealized positions from the molecular geometry and refined isotropically with thermal parameters based on the equivalent displacement parameters of their carriers. Crystallographic data reported in this paper are summarized in Tables S3 and S4. These crystal structures have been deposited to the Cambridge Crystallographic Data Centre.

1.2 Powder X-ray diffraction (PXRD).

Diffractograms were recorded using a PANalytical Empyrean™ diffractometer equipped with a PIXcel^{3D} detector operating in scanning line detector mode with an active length of 4 utilizing 255 channels. The diffractometer is outfitted with an Empyrean Cu LFF (long fine-focus) HR (9430 033 7310x) tube operated at 40 kV and 40 mA and Cu Kα radiation ($\lambda_\alpha = 1.540598$ Å) was used for diffraction experiments. Continuous scanning mode with the goniometer in the theta-theta orientation was used to collect the data. Incident beam optics included the Fixed Divergences slit

with anti-scatter slit PreFIX module, with a 1/8° divergence slit and a 1/4° anti-scatter slit, as well as a 10 mm fixed incident beam mask and a Soller slit (0.04 rad). Divergent beam optics included a P7.5 anti-scatter slit, a Soller slit (0.04 rad), and a Ni-*β* filter. In a typical experiment, 25 mg of sample was dried, ground into a fine powder and was loaded on a zero background silicon disks. The data was collected from 3°-50° (2θ) with a step-size of 0.0131303° and a scan time of 30 seconds per step. Crude data were analyzed using the X'Pert HighScore Plus™ software V 4.1 (PANalytical, The Netherlands).

1.3 Thermogravimetric analysis (TGA)

Thermograms were recorded under N_2 atmosphere using TGA instrument TA Q50 V20.13 Build 39. Aluminium pans and a flow rate of 60 cm^3 min^{-1} for the nitrogen gas were used for the experiments. The data was collected in the High Resolution Dynamic mode with a sensitivity of 1.0, a resolution of 4.0, and a temperature ramp of 10°C min^{-1} up to 550°C.

1.4 In-situ Variable Temperature Powder X-ray Diffraction (VT-PXRD)

Diffractograms at different temperature were recorded using a PANalytical X'Pert Pro-MPD diffractometer equipped with a PIXcel3D detector operating in scanning line detector mode with an active length of 4 utilizing 255 channels. Anton Paar TTK 450 stage coupled with the Anton Paar TCU 110 Temperature Control Unit was used to record the variable temperature diffractograms. The diffractometer is outfitted with an Empyrean Cu LFF (long fine-focus) HR (9430 033 7300x) tube operated at 40 kV and 40 mA and CuKα radiation (λ_α = 1.54056 Å) was used for diffraction experiments. Continuous scanning mode with the goniometer in the theta-theta orientation was used to collect the data. Incident beam optics included the Fixed Divergences slit, with a 1/4° divergence slit and a Soller slit (0.04 rad). Divergent beam optics included a P7.5 anti-scatter slit, a Soller slit (0.04 rad), and a Ni-β filter. In a typical experiment, 20 mg of sample was loaded on a zero background sample holder made for Anton Paar TTK 450 chamber. The data was

collected from 4°-40° (2θ). Crude data were analyzed using the X'Pert HighScore Plus™ software V 4.1 (PANalytical, The Netherlands). The sample was heated up to 350°C under N_2 atmosphere.

1.5 Low Pressure Gas Adsorption Studies

For gas sorption experiments, ultrahigh-purity gases were used as received from BOC Gases Ireland: research-grade CO_2 (99.995%), and N_2 (99.998%). Adsorption experiments (up to 1 bar) were performed on Micromeritics Tristar II 3030 instrument. Before sorption measurements, activation of **sql-4,5-Zn-β** was achieved by degassing the air-dried samples on a SmartVacPrep™ using dynamic vacuum overnight. About 100 mg of activated samples were used for the measurements. A Julabo temperature controller was used to maintain a constant temperature in the bath throughout the experiment. The low temperature at 77 K and 195 K were controlled by a 4 L Dewar filled with liquid N_2 and a mixture of dry ice/acetone, respectively. At every interval of two independent isotherms recorded for any sorbent, samples were regenerated by degassing under high vacuum, before commencing the next sorption experiment.

1.6 Dynamic Vacuum Vapor Sorption

Dynamic vapor sorption measurements were conducted using a Surface Measurement Systems DVS Vacuum at 298 K. Activated samples of **sql-4,5-Zn-β** were further degassed under high vacuum ($1x10^{-4}$ Torr) *in-situ* and stepwise increase in relative pressure were controlled by equilibrated weight changes of the sample (dM/dT = 0.01%/min) from 0 to 95%. Vacuum pressure transducers were used with the ability to measure from $1x10^{-6}$ to 760 Torr with a resolution of 0.01%. Approximately 10 mg of sample was used for each experiment. The mass of the sample was determined by comparison to an empty reference pan and recorded by a high resolution microbalance with a precision of 0.1 μg.

1.7 C_8 aromatics selectivity studies using Nuclear Magnetic Resonance (NMR)

~30 mg samples of **sql-4,5-Zn-β** were separately immersed in equimolar (1 g each) binary/ternary/quaternary liquid of C_8 aromatics at room temperature for one day. PXRD patterns (Figure S2) and TGA curves (Figure 2) reveal that **sql-4,5-Zn-β** completely adsorbed the C_8 aromatics. Then the saturated samples were filtered and air-dried (*ca.* 10 min) under ambient conditions (*ca*. 25 ºC) to remove xylenes adhering to the surface of samples. After that, the samples were soaked in 1 mL DMSO-d_6 for two days.

The supernatant of each sample after soaking in DMSO-d_6 were filtered and collected to measure ^{1}H NMR spectra (JEOL ECX400 NMR spectrometer). The reliability of NMR has been verified in our recent paper.[7] The selectivity is defined as:

$$S_{ij} = \frac{x_i}{x_j}\frac{y_j}{y_i}$$

where *S* is the selectivity of component *i* relative to component *j*, x_i and x_j are the mole fractions of components *i* and *j* in the adsorbed phase, and y_i and y_j are the mole fractions of components *i* and *j* in the liquid phase. For equimolar binary phase, the selectivity can be simplified as

$$S_{ij} = \frac{x_i}{x_j}$$

The ratio of x_i/x_j can be derived from the integrated area ratio of corresponding methyl groups or methylene group of C_8 aromatics in NMR spectra. When component *i* and *j* are both xylene isomers, the selectivity is defined as:

$$S_{ij} = \frac{x_i}{x_j} = \frac{q_i}{q_j}$$

Where q_i and q_j are the relatively integrated area of corresponding methyl groups of xylene isomers. When component *i* is one of xylene isomers while *j* is ethylbenzene, the selectivity is defined as:

$$S_{ij} = \frac{x_i}{x_j} = \frac{q_i}{3q_j}$$

Where q_i is the relatively integrated area of corresponding methyl groups (including 6 H) of xylene isomers, while q_j is the relatively integrated area of corresponding methylene group (including 2 H) of ethylbenzene.

1.8 C_8 aromatics selectivity studies using Gas Chromatography (GC)

Vapour phase: 20 mg samples of **sql-4,5-Zn-β** were separately kept in small vials which stand inside bigger capped vials containing the mixtures of C_8 aromatics at 303 K for three days. Then the saturated samples were air-dried under ambient conditions (*ca*. 25 ºC) to remove xylenes adhering to the surface of samples until the sample is free to move. After that, they were soaked in 1.5 mL CH_2Cl_2 for about three days, allowing C8 aromatics to be completely extracted by CH_2Cl_2. The liquid was collected for GC measurements to calculate the ratios and selectivity coefficients. The samples were also submitted to second extraction in CH_2Cl_2 for GC measurements to make sure the extraction experiments in the first time are complete.

We calculated the saturated vapor pressure (SVP) of C8 aromatics based on Antoine equation under 303K (the equation is presented in supporting information). The values of SVP of C8 aromatics and the ratios of liquid phase are listed below and provided equimolar gaseous phases of C_8 aromatics.

Saturated vapor pressure (SVP) of C_8 aromatics at 303K:

SVP(OX) = 8.85841

SVP(MX) = 11.04598

SVP(PX) = 11.62993

SVP(EB) = 12.61894

The ratios of liquid phase to get equimolar gaseous phases of C_8 aromatics:

Ratio (MX/OX) = 1:1.2470

Ratio (PX/OX) = 1:1.3129

Ratio (EB/OX) = 1:1.4245

Ratio (PX/MX) = 1:1.0529

Ratio (EB/MX) = 1:1.1424

Ratio (EB/PX) = 1:1.0850

Ratio (EB/MX/OX) = 1:1.1424:1.4245

Ratio (PX/MX/OX) = 1:1.0529:1.3129

Ratio (EB/PX/OX) = 1:1.0850:1:1.4245

Ratio (EB/PX/MX) = 1:1.0850:1.1424

Ratio (EB/PX/MX/OX) = 1:1.0850:1.1424:1.4245

Liquid phase: 20 mg samples of **sql-4,5-Zn-β** were separately immersed in equimolar (1 g each) binary/ternary/quaternary liquid of C_8 aromatics at room temperature for one day. Then the saturated samples were filtered and air-dried (*ca.* 10 min) under ambient conditions (*ca*. 25 ºC) to remove xylenes adhering to the surface of samples. After that, the samples were soaked in 1.5 mL CH_2Cl_2 for two days, allowing C8 aromatics to be completely extracted by CH_2Cl_2. The liquid was collected for GC measurements to calculate the ratios and selectivity coefficients. The samples were also extracted by CH_2Cl_2 for the second time for GC measurements to make sure the extraction experiments in the first time are complete. The peak areas of individual C8 aromatics shown in chromatograms were used to calculate the selectivity coefficients by the following method.

The selectivity coefficient of component i relative to component j is defined as:

$$S_{ij} = \frac{x_i y_j}{x_j y_i}$$

where x_i and x_j are the mole fractions of components i and j in the adsorbed phase, and y_i and y_j are the mole fractions of components i and j in the liquid phase. The following equation was used to obtain the adsorption selectivity for quaternary mixture adsorption ($S_{PX/OME}$)

$$S_{PX/OME} = \frac{x_{PX}}{x_{OX} + x_{MX} + x_{EB}} / \frac{y_{PX}}{y_{OX} + y_{MX} + y_{EB}}$$

GC instrument settings: The analyses were carried out on an Agilent 6890A gas chromatograph fitted with a 7683B ALS (automated liquid sampler) equipped with a flame ionization detector (FID). The column used was an Agilent DB-Wax (Length: 30 m, Inner diameter: 320 µm, Film thickness: 0.25 µm). An initial temperature of 40°C and initial hold time of two minutes were used with a ramp rate of 10°C/min to a maximum temperature of 180°C. The injector and detector were kept at 220°C and nitrogen was used as carrier gas with a flow rate of 1 ml/min. 1 µl of each liquid sample was injected through the GC inlet with a split ratio of 100:1 and a split flow rate of 142.45 ml/min. Dichloromethane HPLC/GC grade 99.9% (Sigma-Aldrich) was used as eluent and solvent for the standard solutions of 500 ppm of all the C8 aromatic isomers. With the method described above, we could obtain separate retention times for PX, MX, OX and EB.

1.9 Calculation of the Langmuir surface area

The well-known Langmuir isotherm model can be expressed by the following equation:

$$\frac{Q}{Q_0} = \frac{bP}{1+bP}$$

Where Q/(cm^3 g^{-1}) is the amount adsorbed; Q_0/(cm^3 g^{-1}) is the saturated amount adsorbed; P/mmHg is the equilibrium pressure; and b/$mmHg^{-1}$ is the adsorption affinity.

A line expression for the Langmuir equation can be written as following:

$$\frac{P}{Q} = \frac{1}{bQ_0} + \frac{1}{Q_0}P$$

A least-squares fit is performed on the ($\frac{P}{Q}$, P) designated pairs where $\frac{P}{Q}$ is the independent variable and *P* is the dependent variable. The following are calculated:

a) Slope ($\frac{1}{Q_0}$, g/cm^3 STP)

b) Y-intercept ($\frac{1}{bQ_0}$, g·mmHg/cm^3 STP)

c) Error of the slope (g/cm^3 STP)

d) Error of the y-intercept (g·mmHg/cm^3 STP)

Using the results of the above calculations, the Langmuir surface area can be calculated as following:

$$S_g = A_m \times N_A \times \frac{Q_0}{22414} \times 10^{-18}$$

Where S_g is the Langmuir surface area (m^2/g); A_m = molecular cross-sectional area (nm^2) of adsorbate i.e. 0.1700 nm^2 for CO_2, and $N_A = 6.02 \times 10^{23}$.

1.10 The Antoine equation

For each batch of experiments, the saturated pressure of each xylene isomers was calculated by the following equation:

$$\lg P = A - \frac{B}{t+C}$$

Where P is the pressure of pure component, mmHg. A, B and C were constants and the physical property data, which can be found in various manuals. t was the temperature, ^{o}C. The values of A, B and C were displayed in Table S17.

2. Comparison of C_8 aromatics.

Table S1. Comparison of physicochemical properties for C_8 aromatics.

	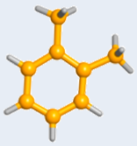	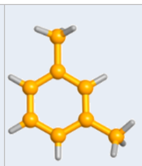	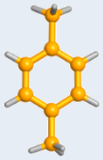	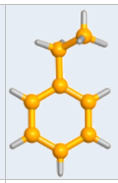
Adsorbate molecules	*ortho*–xylene (OX)	*meta*–xylene (MX)	*para*–xylene (PX)	ethylbenzene (EB)
Molecular dimension (Å^3)	3.83 ×7.27 ×7.83	3.95 ×7.32 ×8.99	3.28 ×6.63 ×7.34	5.29 ×6.63 ×9.36
Kinetic diameter (Å)	6.8	6.8	5.8	5.8
F.P. (K)	249	225	286.3	178.2
B.P. (K)	417.6	412.3	411.5	409.3
Dipolar moment	0.62	0.36	0.1	0.59
Polarizability σ (x10^{-25}, cm^3)	141-149	142	137-149	142

3. CSD search results

Survey of crystallographic and topological databases

The list of MOMs having **sql** net topology was obtained from the TTO TOPOS database[8] (version: Dec 2021); valence-bonded MOFs in standard representation were used. The listed MOM crystal structures from the TTO database were analyzed using to the Cambridge Structural Database (CSD version 5.43, March 2022) through ConQuest software.[9]

Table S2. A summary of reported bent dicarboxylate ligand based **sql** networks assembled from parallel layers with paddlewheel units found in the database survey.

RefCode	Formula	Interpenetration?	DOI	Properties	Organic linkers
**sql-4,5-Zn-α**	**$C_{51}H_{37}N_5O_{13}S_2Zn_2$**	**Non-interpenetrated**	**This work**	**C_8 aromatics separation**	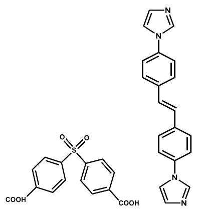
**sql-4,5-Zn-β**	**$C_{24}H_{16}N_2O_6SZn$**				
**sql-4,5-Zn·2OX**	**$C_{64}H_{52}N_4O_{12}S_2Zn_2$**				
**sql-4,5-Zn·2MX**	**$C_{64}H_{52}N_4O_{12}S_2Zn_2$**				
**sql-4,5-Zn·2PX**	**$C_{64}H_{52}N_4O_{12}S_2Zn_2$**				
**sql-4,5-Zn·2EB**	**$C_{64}H_{52}N_4O_{12}S_2Zn_2$**				
**sql-4,5-Zn·1MX**	**$C_{56}H_{42}N_4O_{12}S_2Zn_2$**				
**sql-4,5-Zn·1PX**	**$C_{56}H_{42}N_4O_{12}S_2Zn_2$**				
**sql-4,5-Zn·1EB**	**$C_{56}H_{42}N_4O_{12}S_2Zn_2$**				
AGOMAN	$(C_{40}H_{26}N_4O_{12}S_2Zn_2)_n$, $2n(C_3H_7NO)$	Interpenetrated	10.1039/C8NJ03302A	Photoluminescence	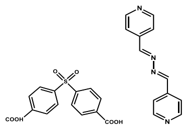
JUYPAX	$(C_{40}H_{26}Cu_2N_4O_{12}S_2)_n$		10.1016/j.molstruc.2020.129357	Salpingitis treatment	
SEPMEI	$(C_{40}H_{26}Co_2N_4O_{12}S_2)_n$, C_3H_7NO, $4(H_2O)$		10.1021/acs.inorgchem.7b02136	CO_2 separation	
KUJQAK	$(C_{42}H_{30}Co_2N_4O_{12}S_2)_n$	Non-interpenetrated	CSD Communication(Private Communication) (2020)	-	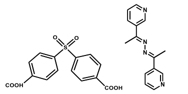
DEBTAI	$(C_{38}H_{24}Co_2N_2O_{12}S_2)_n$, $2(H_2O)$	Interpenetrated	10.1039/C7RA07637A	Magnetism	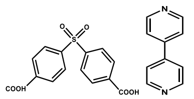
KEZPIP	$(C_{38}H_{24}Cu_2N_2O_{12}S_2)_n$, $2n(H_2O)$		10.1016/j.poly.2006.10.007	NG	
RIHJID	$(C_{38}H_{24}N_2O_{12}S_2Zn_2)_n$		10.1021/ic302006x	Flexibility	
RUBGIF	$(C_{38}H_{24}N_2Ni_2O_{12}S_2)_n$, $2n(H_2O)$	Interpenetrated	10.1016/j.solidstatesciences.2008.06.011	NG	
SULKEQ	$(C_{38}H_{24}N_2O_{12}S_2Zn_2)_n$, $2n(H_2O)$	Interpenetrated	Chin.J.Struct.Chem.) (2009), 28, 1417	Photoluminescence	
GOVXAS	$(C_{48}H_{42}Co_2N_4O_{10})_n$, $2n(CH_4O)$	Interpenetrated	10.1039/C4CE02101K	CO_2 adsorption	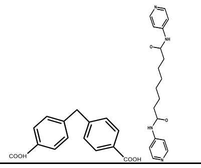
RESWET	$(C_{46}H_{38}N_4Ni_2O_{14}S_2)_n$, $6n(H_2O)$	Interpenetrated	10.1039/c3ce26928k	Magnetism	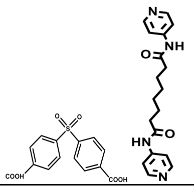

SUDTUJ	$(C_{46}H_{38}Co_2N_4O_{14}S_2)_n$, $n(CH_4O)$, $n(H_2O)$	Interpenetrated	10.3390/molecules25010201	Magnetism	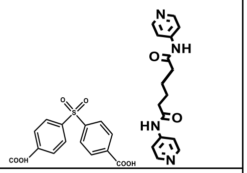
PITJEK	$(C_{40}H_{26}N_4O_{13}S_2Zn_2)_n$	Non-interpenetrated	10.1039/C8CE01925H	CO_2 separation	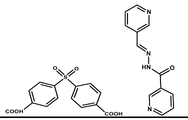
RIHKAW	$(C_{44}H_{28}N_2O_{12}S_2Zn_2)_n$, $2n(C_3H_7NO)$	Non-interpenetrated	10.1021/ic302006x	Flexibility	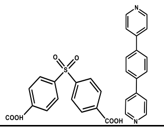
RIHJOJ	$(C_{40}H_{24}N_6O_{12}S_2Zn_2)_n$, $2(C_3H_7NO)$	Non-interpenetrated	10.1021/ic302006x	Flexibility	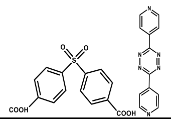
RIHKEA	$(C_{34}H_{28}N_2O_{12}S_2Zn_2)_n$	Non-interpenetrated	10.1021/ic302006x	Flexibility	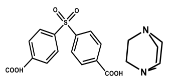
YEPLIR	$(C_{66}H_{40}N_2O_8Zn_2)_n$, $n(C_3H_7NO)$, $3.5n(H_2O)$	Non-interpenetrated	10.1021/acs.cgd.7b01392	Photoluminescence	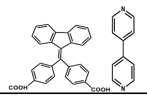
ASOFIZ	$(C_{48}H_{32}Co_2N_2O_{12}S_2)_n$	Non-interpenetrated	10.1021/acs.cgd.6b01431	Magnetism	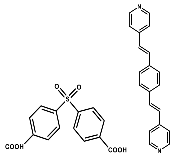
ASOFOF	$(C_{48}H_{32}Co_2N_2O_{12}S_2)_n$, C_3H_7NO	Interpenetrated	10.1021/acs.cgd.6b01431	Magnetism	
ASOFOF01	$(C_{48}H_{32}Co_2N_2O_{12}S_2)_n$,$3(C_3H_7NO)$				
ASOFUL	$(C_{48}H_{32}Co_2N_2O_{12}S_2)_n$,$C_4H_9NO$, $2(H_2O)$				
ASOGEW	$(C_{48}H_{32}Co_2N_2O_{12}S_2)_n$,$C_6H_4Cl_2$				
ASOGIA	$(C_{48}H_{32}Co_2N_2O_{12}S_2)_n$,$C_6H_4Br_2$				
HOFPUP	$(C_{48}H_{32}N_2O_{12}S_2Zn_2)_n$,	Interpenetrated	10.1002/anie.201310536	[2+2] cycloaddition reaction	
HOFQAW	$(C_{48}H_{32}N_2O_{12}S_2Zn_2)_n$,$0.5n(C_2H_6OS)$				
HOFQEA	$(C_{48}H_{32}N_2O_{12}S_2Zn_2)_n$	Non-interpenetrated			
HECYIZ	$(C_{42}H_{30}Co_2N_4O_{12}S_2)_n$	Non-interpenetrated	10.1021/ic301636z	Photoluminescence	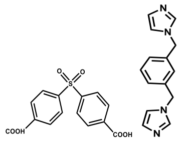
HECYUL	$(C_{42}H_{30}Cd_2N_4O_{12}S_2)_n$				
HECYOF	$(C_{42}H_{30}Zn_2N_4O_{12}S_2)_n$				
VADJES	$(C_{42}H_{30}Co_2N_4O_{12}S_2)_n$, $4n(H_2O)$	Non-interpenetrated	10.1039/C6DT00349D	NG	
HIYJAC	$(C_{45}H_{36}N_4O_{12}S_2Zn_2)_n$, $2n(C_4H_8O)$	Non-interpenetrated	10.1016/j.jssc.2014.02.007	Dye degradation	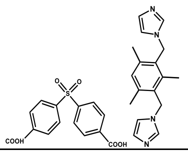
HIYJEG	$(C_{45}H_{36}Co_2N_4O_{12}S_2)_n$, $3n(C_4H_8O_2)$				

UXOLIE	$(C_{52}H_{38}Cd_2N_4O_{12}S_2)_n$, H_2O	Non-interpenetrated	10.1515/ncrs-2016-0018	NG	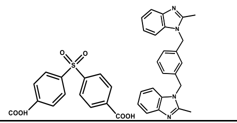
OTIHOR	$(C_{48}H_{42}N_4O_{12}S_2Zn_2)_n$	Interpenetrated	10.1021/acs.cgd.1c00152	Dye degradation	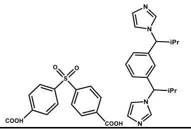
TAHNIB	$(C_{52}H_{36}N_6O_{12}S_2Zn_2)_n$, $n(H_2O)$	Interpenetrated	10.1016/j.ica.2010.07.047	Luminescence	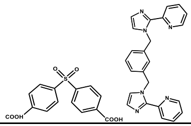
KINHEW	$(C_{46}H_{28}N_2O_{12}S_4Zn_2)_n$	Interpenetrated	10.1021/cg400490y	Photoluminescence	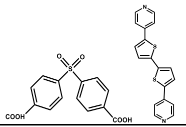
OVEFOM02	$(C_{50}H_{36}Co_2N_2O_{14}S_2)_n$, $2n(C_3H_7NO)$	Non-interpenetrated	10.1039/C6DT03086F	Gas adsorption	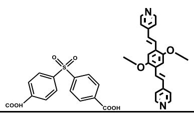
PEHJIY	$(C_{42}H_{30}N_4O_{12}S_2Zn_2)_n$	Non-interpenetrated	10.1039/C7DT03341A	CO_2 separation	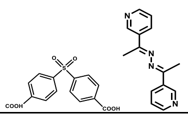
QEQWOB	$(C_{48}H_{30}N_4O_{12}S_3Zn_2)_n$	Interpenetrated	10.1039/C7CE01933E	Dye degradation	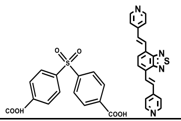
VUPWOV	$(C_{38}H_{24}N_4O_{12}S_2Zn_2)_n$	Non-interpenetrated	10.1021/acs.inorgchem.0c00551	CO_2 separation	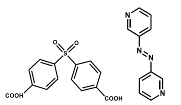
VUPWUB	$(C_{38}H_{24}N_4O_{12}S_2Zn_2)_n$	Interpenetrated			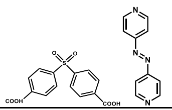
XETGAG	$(C_{44}H_{36}Co_2N_4O_{12}S_2)_n$, $3n(C_4H_8O)$	Non-interpenetrated	10.1021/cg301329c	Luminescence	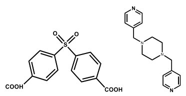
XETGEK	$(C_{44}H_{36}Co_2N_4O_{12}S_2)_n$, $2n(C_4H_8O)$				
OMAHAN	$(C_{36}H_{32}N_2O_8Zn_2)_n$	Interpenetrated	10.11862/CJIC.2016.104	Luminescence	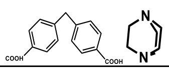
OVAHUQ	$(C_{42}H_{32}Cd_2N_2O_8)_n$, $4(H_2O)$	Interpenetrated	10.1007/s11243-016-0044-5	NG	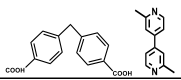
ZUSRIQ	$(C_{42}H_{36}Mn_2N_2O_8Si_2)_n$	Interpenetrated	10.1039/C5DT02354H	Magnetism	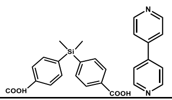
ZUSRIQ01					

4. Crystallographic Data

Table S3. Crystallographic data and refinement parameters of **sql-4,5-Zn-α** and **sql-4,5-Zn-β**.

	**sql-4,5-Zn-α**	**sql-4,5-Zn-β**
CCDC No	2191665	2191666
Formula	$C_{51}H_{37}N_5O_{13}S_2Zn_2$	$C_{24}H_{16}N_2O_6SZn$
Mr	1122.71	525.82
T [K]	150	100
Crystal system	triclinic	triclinic
Space group	*P*-1	*P*-1
a [Å]	11.9087(6)	7.6850(3)
b [Å]	13.1041(7)	13.0434(5)
c [Å]	20.9080(10)	13.6177(5)
α[°]	78.518(2)	70.297(2)
β[°]	76.660(2)	76.892(2)
γ[°]	89.502(2)	75.236(2)
V [Å^3]	3108.8(3)	1227.70(8)
Z	2	2
ρ_c[gcm^{-3}]	1.199	1.422
μ[mm^{-1}]	0.895	2.541
Radiation	Mo Kα	Cu Kα
reflns coll.	66512	55726
unique reflns	15285	4166
GOF	1.073	1.07
R_1[I>2σ(I)][a]	0.0435	0.0478
wR_2[I>2σ(I)][b]	0.1327	0.129

[a] $R_1 = \Sigma||F_o| - |F_c||/\Sigma|F_o|$. [b] $wR_2 = \{\Sigma[w(F_o^2 - F_c^2)^2]/\Sigma[w(F_o^2)^2]\}^{1/2}$.

Table S4. Crystallographic data and refinement parameters of **sql-4,5-Zn·2OX**, **sql-4,5-Zn·2MX**, **sql-4,5-Zn·2PX**, and **sql-4,5-Zn·2EB**.

	**sql-4,5-Zn·2OX**	**sql-4,5-Zn·2MX**	**sql-4,5-Zn·2PX**	**sql-4,5-Zn·2EB**
CCDC No	2191667	2191668	2191669	2191670
Formula	$C_{48}H_{32}N_4O_{12}S_2Zn_2\cdot 2(C_8H_{10})$	$C_{48}H_{32}N_4O_{12}S_2Zn_2\cdot 2(C_8H_{10})$	$C_{48}H_{32}N_4O_{12}S_2Zn_2\cdot 2(C_8H_{10})$	$C_{64}H_{52}N_4O_{12}S_2Zn_2\cdot 2(C_8H_{10})$
Mr	1263.95	1263.95	1263.95	1263.95
T [K]	107	107	107	107
Crystal system	triclinic	triclinic	triclinic	triclinic
Space group	*P*-1	*P*-1	*P*-1	*P*-1
a [Å]	11.6989(6)	11.8088(6)	11.7782(8)	11.9147(6)
b [Å]	13.3563(7)	13.2892(6)	13.2918(8)	13.1195(5)
c [Å]	20.8790(11)	20.5594(10)	20.4698(14)	20.5263(8)
α[°]	76.931(2)	79.141(2)	100.493(2)	79.293(3)
β[°]	74.669(2)	77.702(2)	102.986(3)	76.985(4)
γ[°]	89.974(2)	89.569(2)	90.011(2)	89.902(3)
V [$Å^3$]	3058.7(3)	3094.1(3)	3067.6(4)	3069.0(2)
Z	2	2	2	2
ρ_c[gcm^{-3}]	1.372	1.357	1.368	1.368
μ[mm^{-1}]	0.917	0.906	0.914	0.914
Radiation	Mo Kα	Mo Kα	Mo Kα	Mo Kα
reflns coll.	106325	138199	89343	130256
unique reflns	15109	15461	15300	14771
GOF	1.022	1.041	1.022	1.045
R_1[I>2σ(I)][a]	0.0759	0.0561	0.0723	0.0685
wR_2[I>2σ(I)][b]	0.2225	0.1696	0.1955	0.2071

[a] $R_1 = \Sigma||F_o| - |F_c||/\Sigma|F_o|$. [b] $wR_2 = \{\Sigma[w(F_o^2 - F_c^2)^2]/\Sigma[w(F_o^2)^2]\}^{1/2}$.

Table S5. Crystallographic data and refinement parameters of **sql-4,5-Zn·1MX**, **sql-4,5-Zn·1PX**, and **sql-4,5-Zn·1EB**.

	**sql-4,5-Zn·1MX**	**sql-4,5-Zn·1PX**	**sql-4,5-Zn·1EB**
CCDC No	2217609	2217610	2217611
Formula	$C_{48}H_{32}N_4O_{12}S_2Zn_2\cdot C_8H_{10}$	$C_{48}H_{32}N_4O_{12}S_2Zn_2\cdot C_8H_{10}$	$C_{48}H_{32}N_4O_{12}S_2Zn_2\cdot C_8H_{10}$
Mr	1157.79	1157.79	1157.79
T [K]	100.00	100.00	100.00
Crystal system	triclinic	triclinic	triclinic
Space group	*P*-1	*P*-1	*P*-1
a [Å]	11.7742(17)	11.8650(12)	11.8391(12)
b [Å]	12.9823(16)	12.6946(12)	12.7930(13)
c [Å]	18.814(3)	18.7784(16)	18.9102(16)
α[º]	74.112(5)	105.581(8)	104.937(8)
β[º]	89.079(6)	90.276(8)	90.067(7)
γ[º]	73.721(4)	105.272(9)	105.206(9)
V [$Å^3$]	2649.4(6)	2619.6(5)	2663.2(5)
Z	2	2	2
$\rho_c[gcm^{-3}]$	1.451	1.468	1.444
$\mu[mm^{-1}]$	1.051	1.063	1.046
Radiation	MoKα	Mo Kα	Mo Kα
reflns coll.	70034	35224	44232
unique reflns	8260	8969	8576
GOF	1.037	0.979	0.99
$R_1[I>2\sigma(I)]$[a]	0.1040	0.0972	0.0949
$wR_2[I>2\sigma(I)]$[b]	0.2637	0.2429	0.2395

[a] $R_1 = \Sigma||F_o| - |F_c||/\Sigma|F_o|$. [b] $wR_2 = \{\Sigma[w(F_o^2 - F_c^2)^2]/\Sigma[w(F_o^2)^2]\}^{1/2}$.

5. Void volume

Table S6. The void volume of large pore phase, narrow pore phase, fully guest-loaded phases, and partially guest-loaded phases, calculated using PLATON.

Compound	Void volume	Compound	Void volume
**sql-4,5-Zn-α**	39.2%	**sql-4,5-Zn-β**	15.4%
**sql-4,5-Zn·2OX**	37.7%	**sql-4,5-Zn·1OX**	-
**sql-4,5-Zn·2MX**	39.1%	**sql-4,5-Zn·1MX**	25.2%
**sql-4,5-Zn·2PX**	38.3%	**sql-4,5-Zn·1PX**	25.1%
**sql-4,5-Zn·2EB**	38.4%	**sql-4,5-Zn·1EB**	25.6%

6. Powder X-ray Diffraction

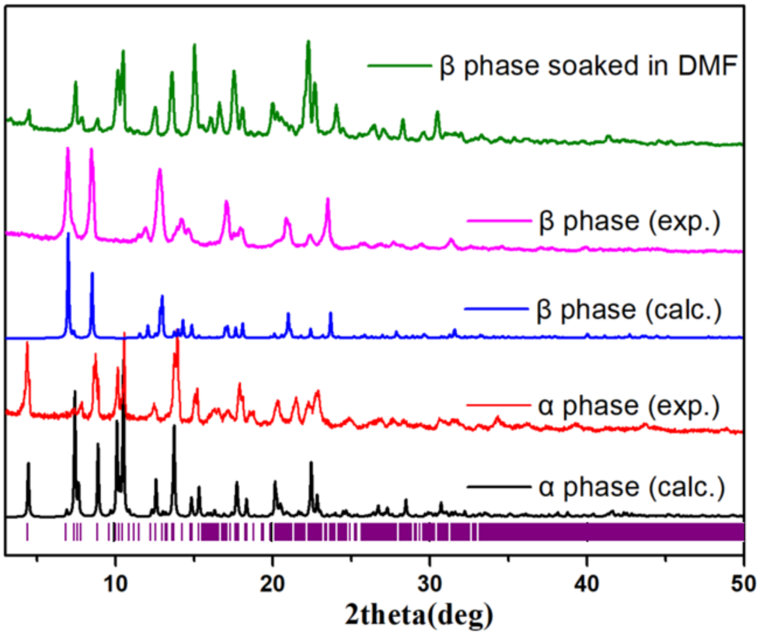

Figure S1. PXRD patterns of **sql-4,5-Zn-α**, **sql-4,5-Zn-β**, and **sql-4,5-Zn-β** after soaking in DMF for two days.

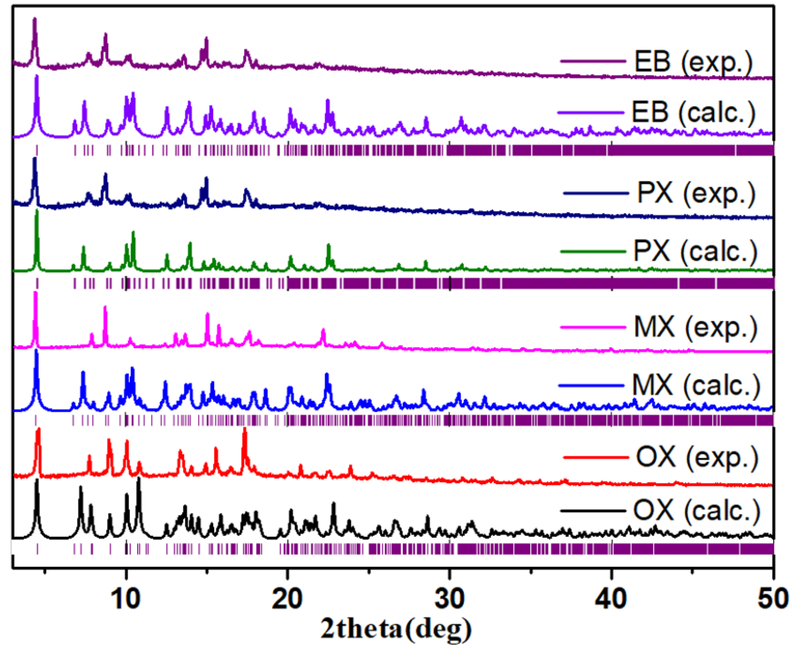

Figure S2. PXRD patterns of **sql-4,5-Zn·2OX**, **sql-4,5-Zn·2MX**, **sql-4,5-Zn·2PX**, and **sql-4,5-Zn·2EB**.

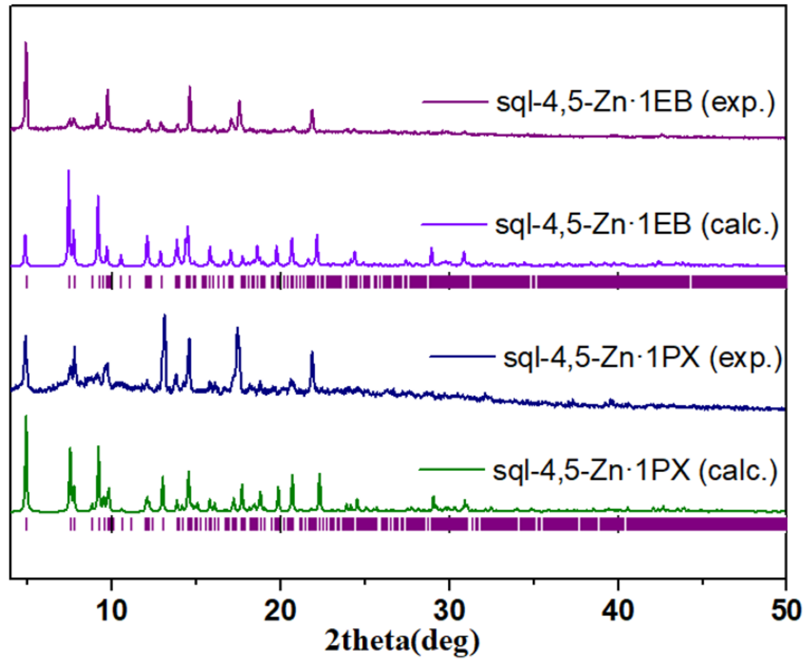

Figure S3. PXRD patterns of **sql-4,5-Zn·1PX** and **sql-4,5-Zn·1EB**.

7. Thermogravimetric analysis (TGA)

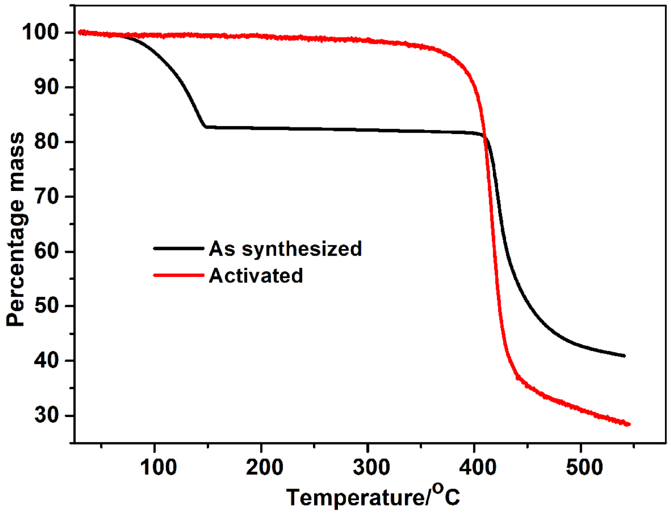

Figure S4. Thermogravimetric analysis profiles of the as-synthesized sample **sql-4,5-Zn-α** and activated sample **sql-4,5-Zn-β** under N_2 environment.

8. VT-PXRD

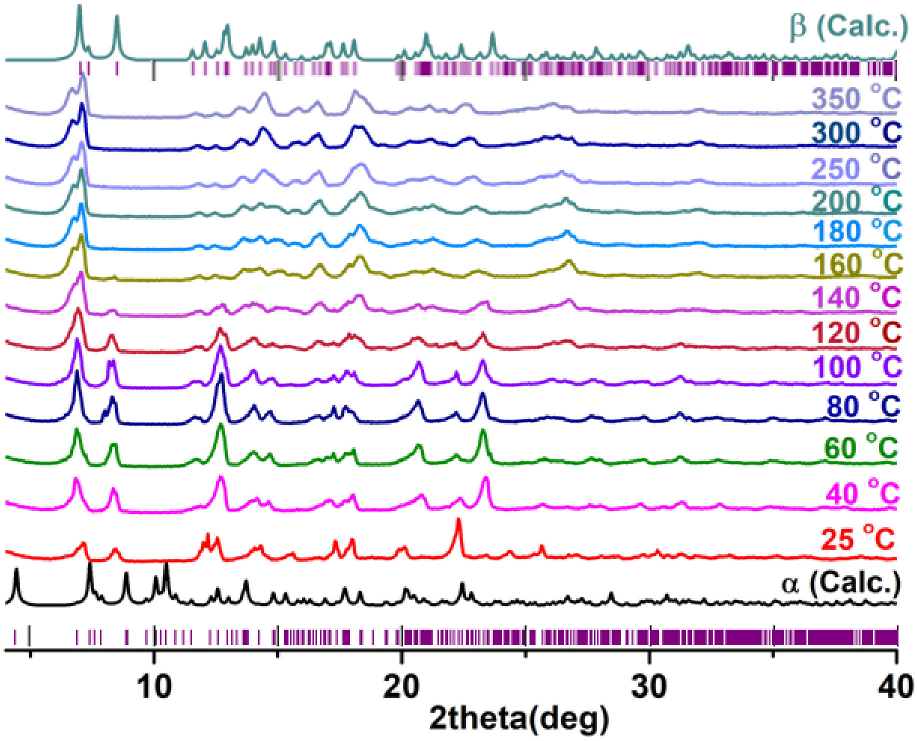

Figure S5. In-situ variable temperature PXRD patterns for **sql-4,5-Zn**.

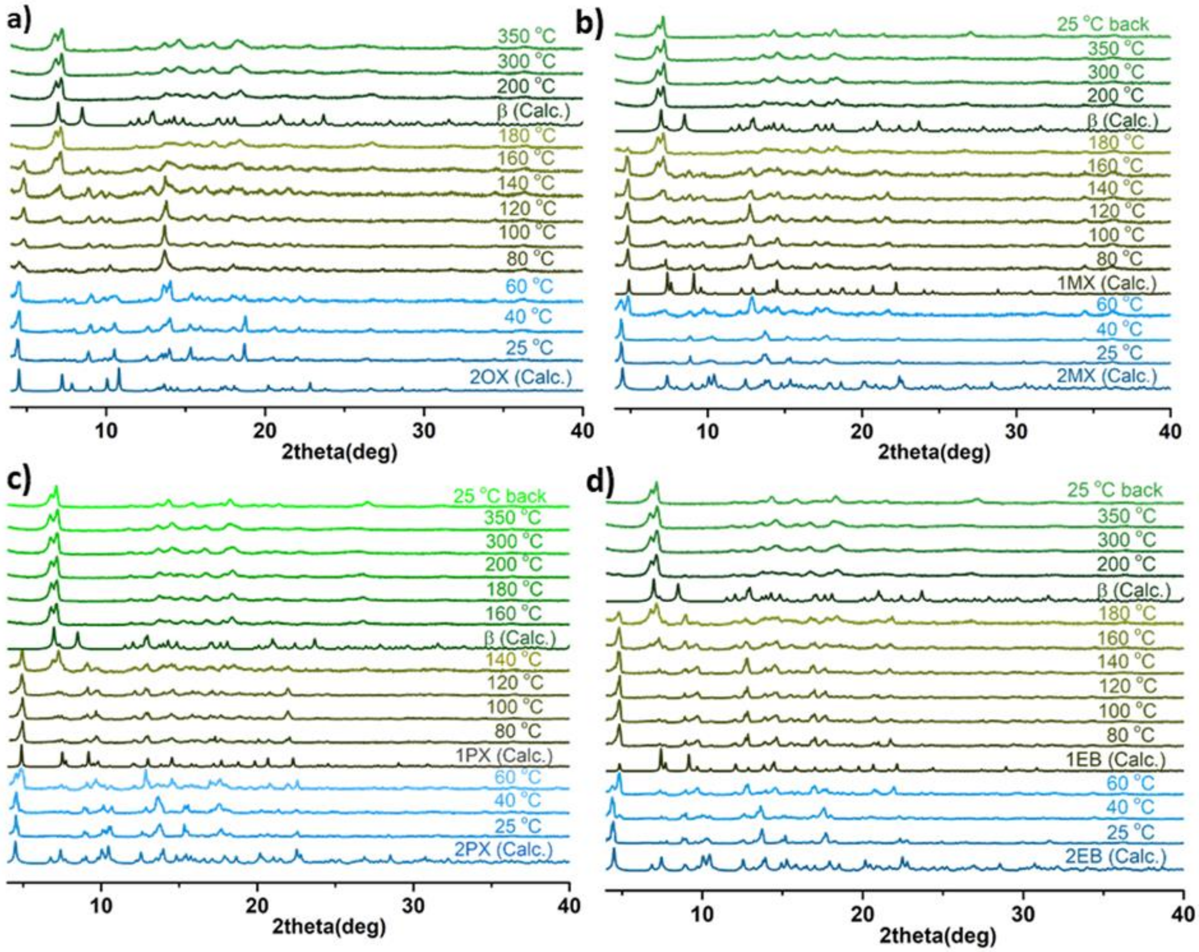

Figure S6. Variable temperature PXRD patterns for **sql-4,5-Zn·2OX** (a), **sql-4,5-Zn·2MX** (b), **sql-4,5-Zn·2PX** (c), and **sql-4,5-Zn·2EB** (d) temperature in °C.

9. Paddle-wheel unit

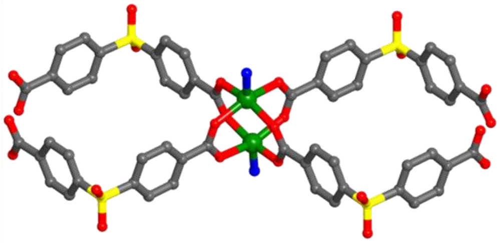

Figure S7. Paddle-wheel unit in rhombohedral grid in **sql-4,5-Zn**.

10. CSD Survey of N-donor ligand

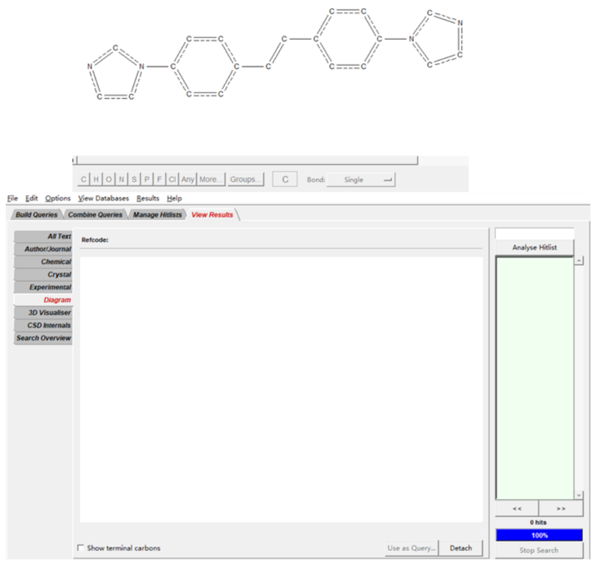

Figure S8. Cambridge Structural Database (CSD) result of trans-4,4′-bis(1-imidazolyl)stilbene with no entry in the Cambridge Structural Database (CSD, v. 5.43, updated to March 2022).[10-12]

11.Comparison of Bond length/Distances and Angle for Crystal Structures

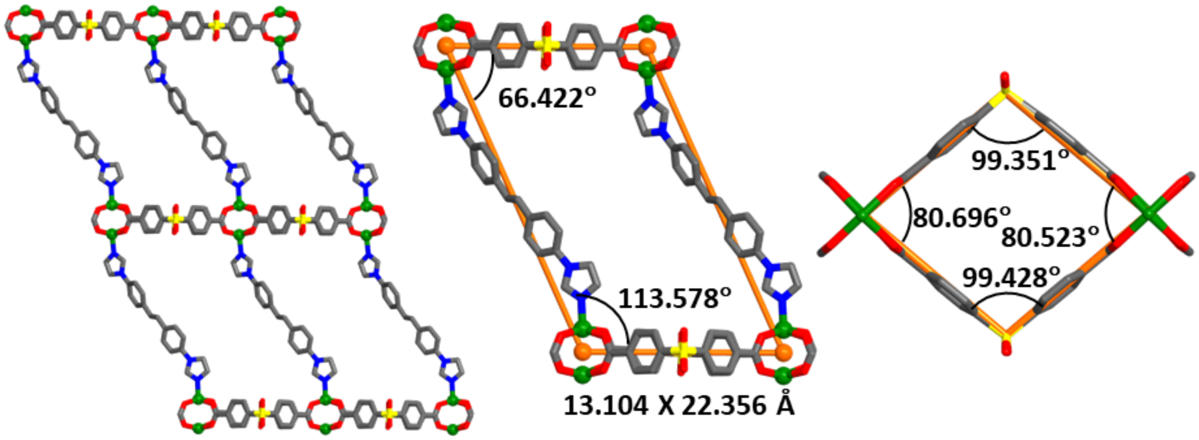

Figure S9. Crystal structure of **sql-4,5-Zn-α** depicting the square grids and layer packing.

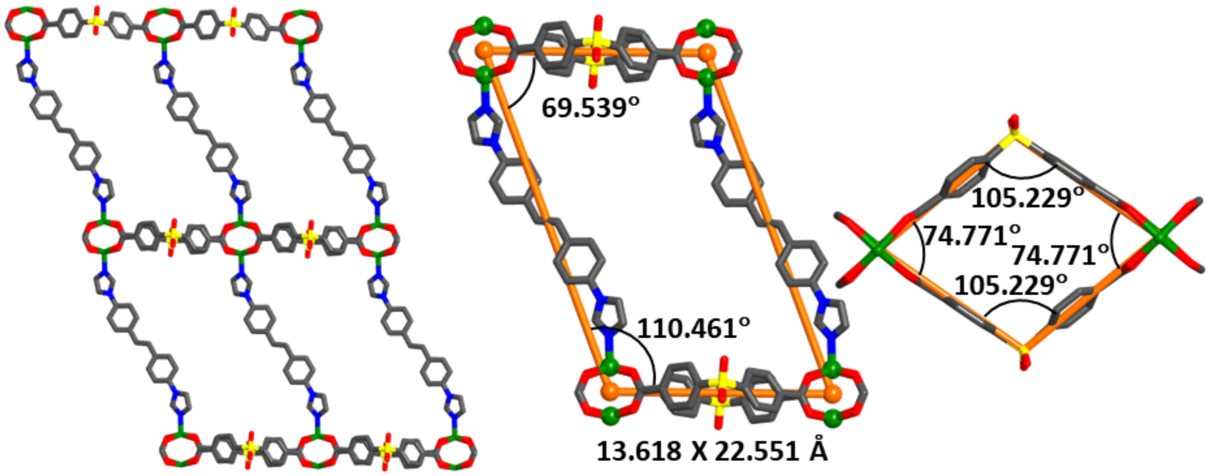

Figure S10. Crystal structure of **sql-4,5-Zn-β** depicting the square grids and layer packing.

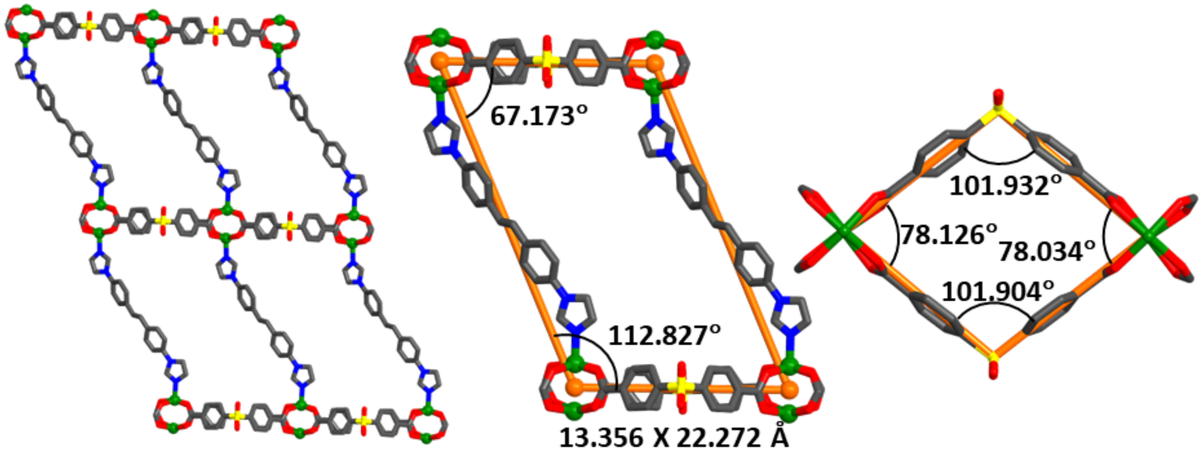

Figure S11. Crystal structure of **sql-4,5-Zn·2OX** depicting the square grids and layer packing.

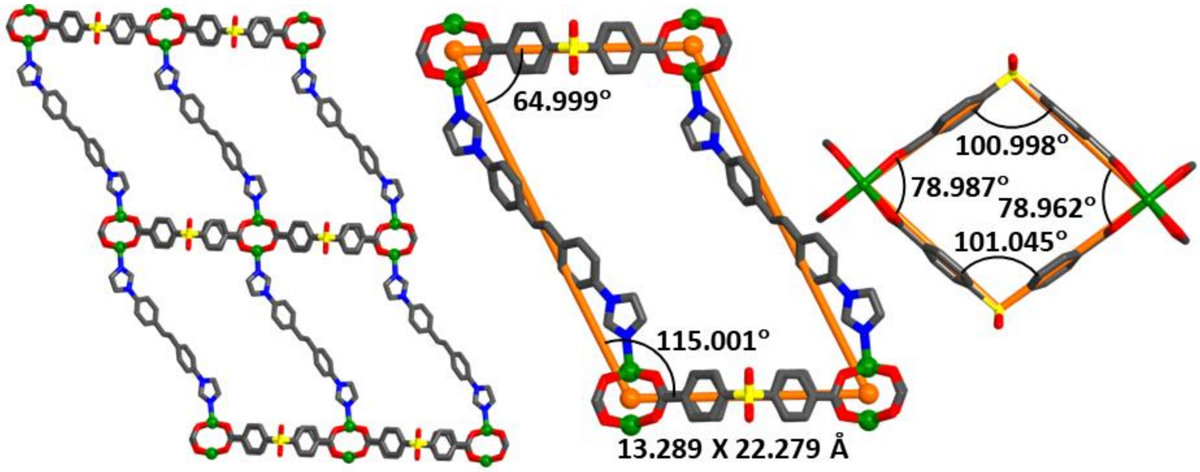

Figure S12. Crystal structure of **sql-4,5-Zn·2MX** depicting the square grids and layer packing.

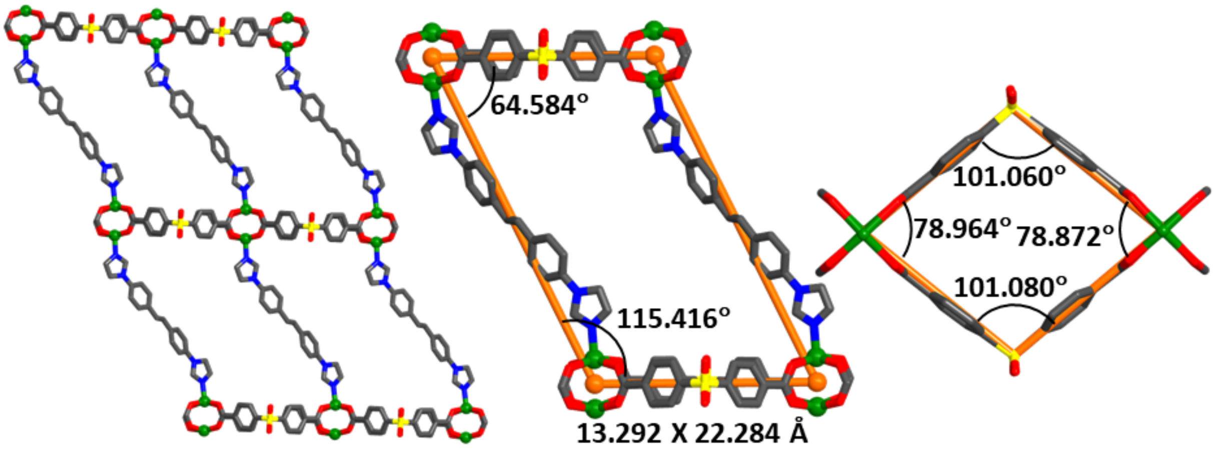

Figure S13. Crystal structure of **sql-4,5-Zn·2PX** depicting the square grids and layer packing.

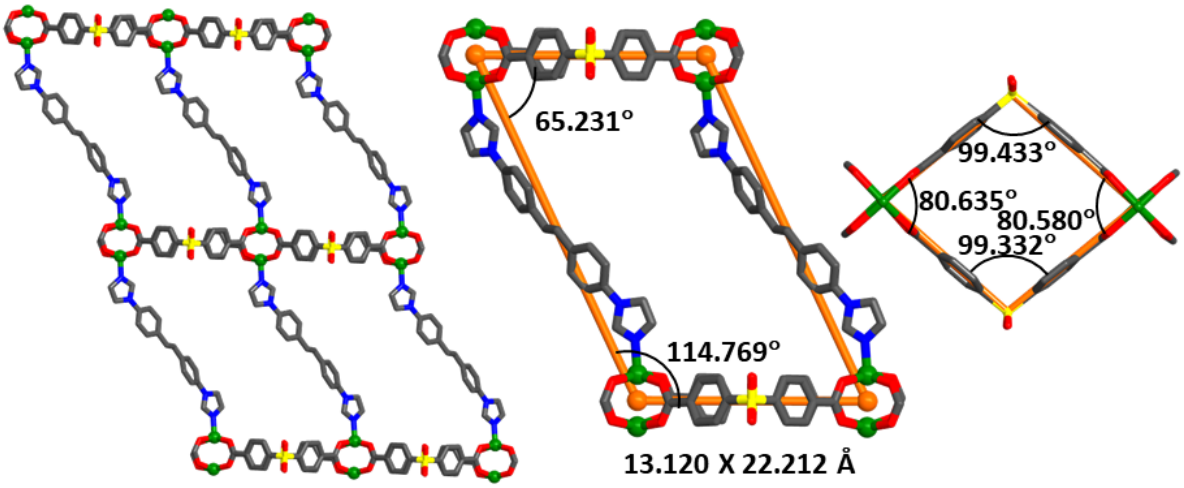

Figure S14. Crystal structure of **sql-4,5-Zn·2EB** depicting the square grids and layer packing.

12. Angles of the Rings in Ditopic N-donor Ligand

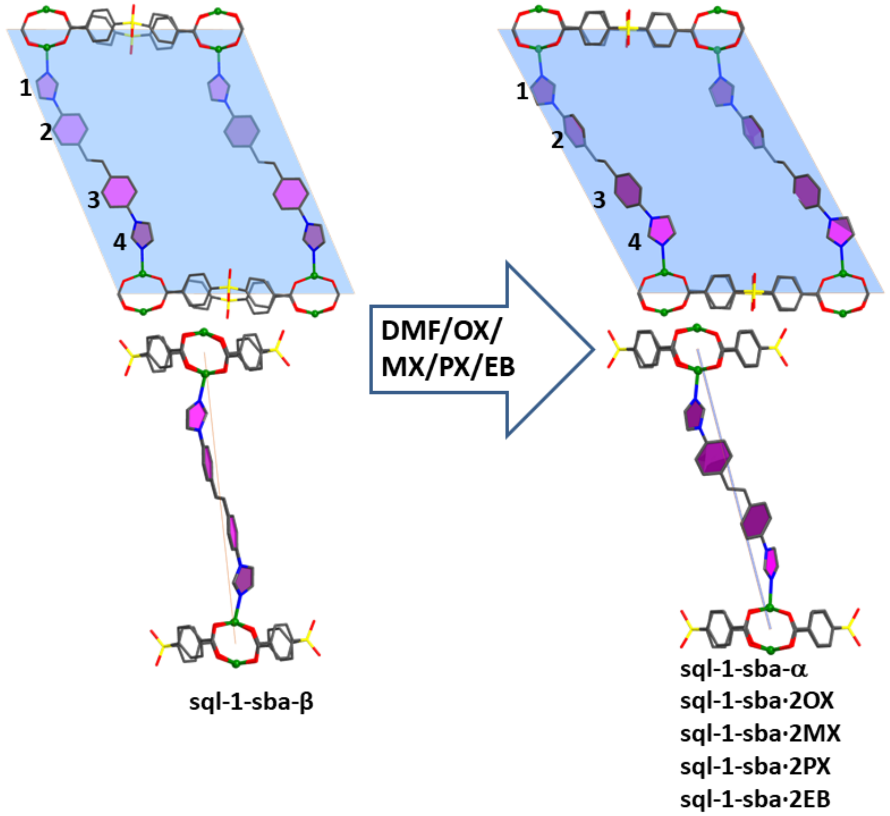

Figure S15. Rotation of the rings in the ditopic N-donor ligand for guest free phase of **sql-4,5-Zn-β** and the porous (LP) phase of **sql-4,5-Zn-α**, **sql-4,5-Zn·2OX**, **sql-4,5-Zn·2MX**, **sql-4,5-Zn·2PX**, and **sql-4,5-Zn·2EB**.

Table S7. Dihedral angles between the aromatic rings of ditopic N-donor ligand and plane of square grid of **sql-4,5-Zn-α**, **sql-4,5-Zn-β**, **sql-4,5-Zn·2OX**, **sql-4,5-Zn·2MX**, **sql-4,5-Zn·2PX** and **sql-4,5-Zn·2EB**.

Compound	Plane 1 (°)	Plane 2 (°)	Plane 3 (°)	Plane 4 (°)
**sql-4,5-Zn-β**	42.869	11.972	11.972	42.869
**sql-4,5-Zn-α**	40.493	70.845	48.4888	15.411
**sql-4,5-Zn·2OX**	36.641	65.254	53.408	22.795
**sql-4,5-Zn·2MX**	41.814	69.426	54.360	27.772
**sql-4,5-Zn·2PX**	40.570	69.309	53.068	22.141
**sql-4,5-Zn·2EB**	41.794	73.960	51.389	20.841

13.Horizontal shift of the layers

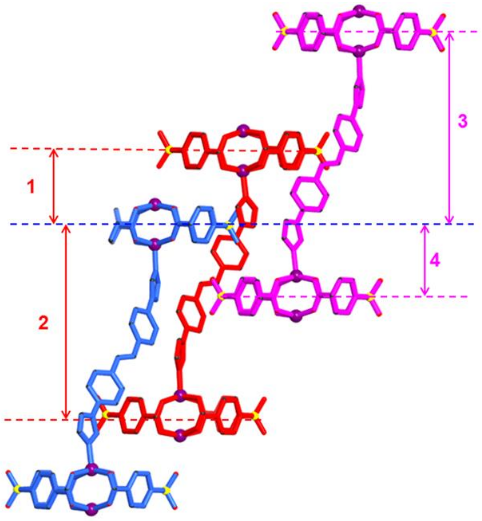

Figure S16. Horizontal shift (Å) of the layers for **sql-4,5-Zn-α**, **sql-4,5-Zn-β**, **sql-4,5-Zn·2OX**, **sql-4,5-Zn·2MX**, **sql-4,5-Zn·2PX** and **sql-4,5-Zn·2EB**.

Table S8. Horizontal shift (Å) of the layers for **sql-4,5-Zn-α**, **sql-4,5-Zn-β**, **sql-4,5-Zn·2OX**, **sql-4,5-Zn·2MX**, **sql-4,5-Zn·2PX** and **sql-4,5-Zn·2EB**.

	**1 (Å)**	**2 (Å)**	**3 (Å)**	**4 (Å)**
**sql-4,5-Zn-α**	5.6043	14.3680	14.6742	5.2981
**sql-4,5-Zn-β**	5.8860	15.2052	11.7719	9.3192
**sql-4,5-Zn·2OX**	9.1867	11.0870	15.0613	5.2125
**sql-4,5-Zn·2MX**	5.5934	14.0032	14.3248	5.2718
**sql-4,5-Zn·2PX**	5.6311	13.9522	14.2710	5.3123
**sql-4,5-Zn·2EB**	5.5271	14.1308	14.3642	5.2937

14. Sorption Isotherms

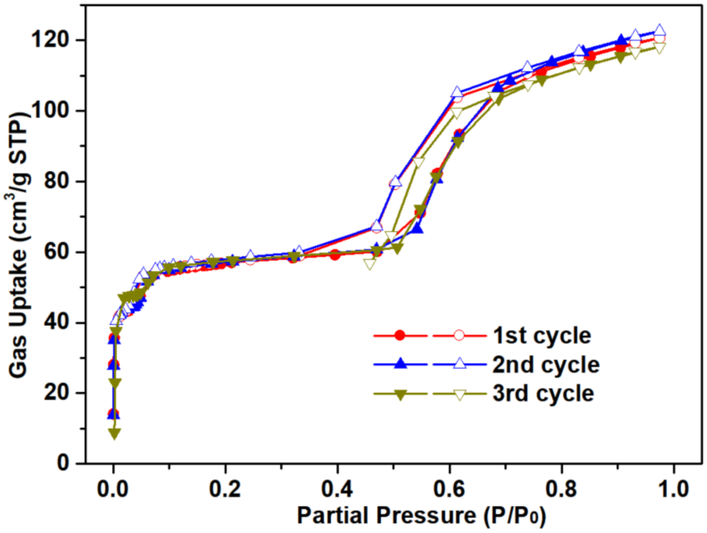

Figure S17. CO_2 isotherms at 195 K for the activated **sql-4,5-Zn-β**, recorded over three consecutive cycles.

15. ^{1}H NMR Spectra.

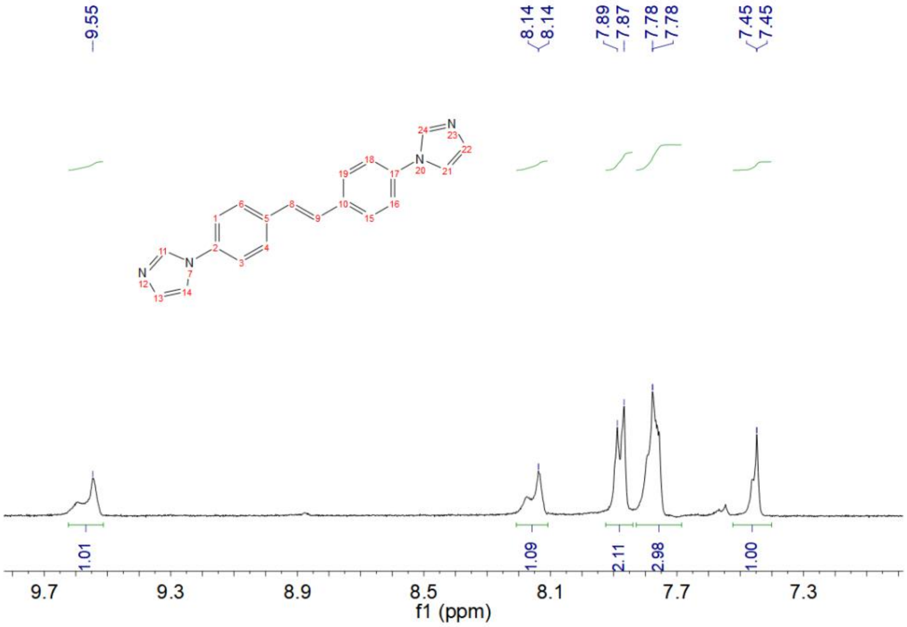

Figure S18. ^{1}H NMR (400 MHz, $(CD_3)_2SO$) of trans-4,4′-bis(1-imidazolyl)stilbene.

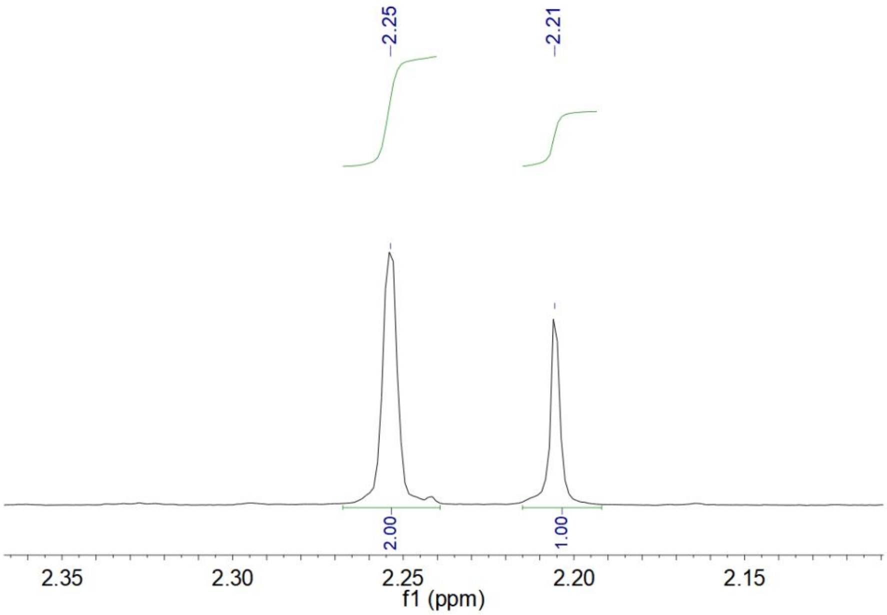

Figure S19. Magnified ^{1}H NMR spectrum recorded using the DMSO-d$_6$ extract of C_8 aromatics obtained from **sql-4,5-Zn** that was prior subjected to the equimolar binary liquid of MX/OX until saturated.

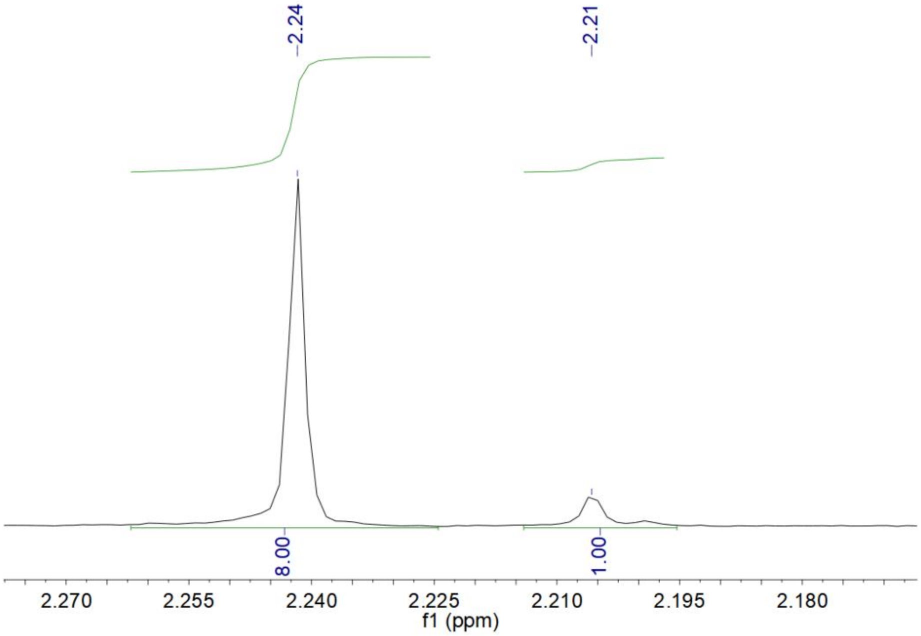

Figure S20. Magnified ^{1}H NMR spectrum recorded using the DMSO-d$_6$ extract of C_8 aromatics obtained from **sql-4,5-Zn** that was prior subjected to the equimolar binary liquid of PX/OX until saturated.

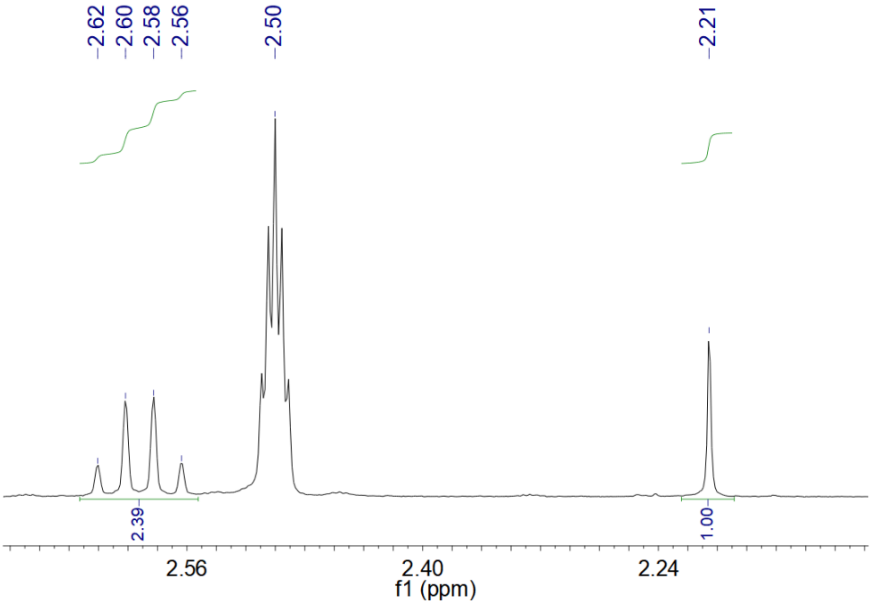

Figure S21. Magnified ^{1}H NMR spectrum recorded using the DMSO-d_6 extract of C_8 aromatics obtained from **sql-4,5-Zn** that was prior subjected to the equimolar binary liquid of EB/OX until saturated.

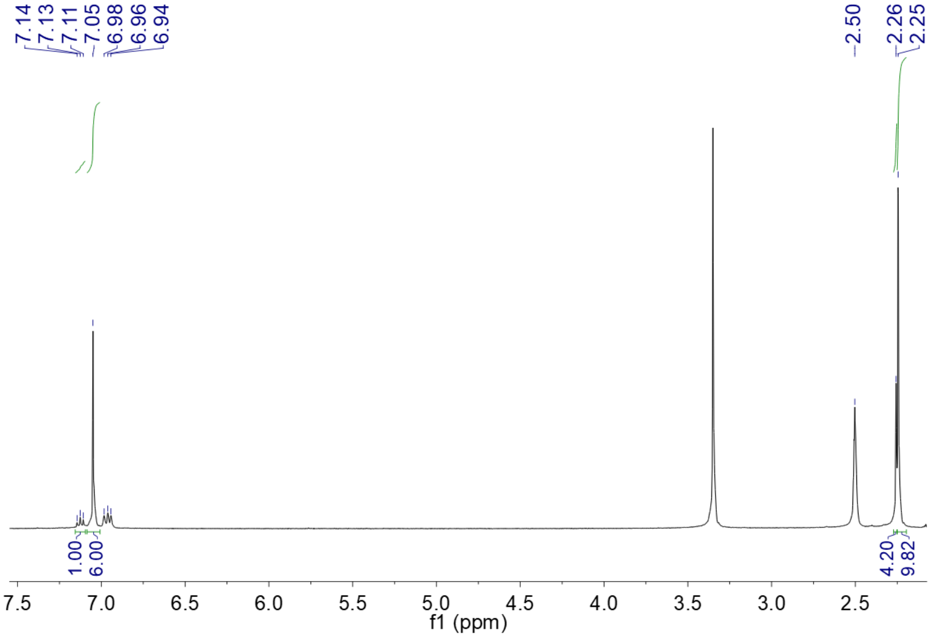

Figure S22. Magnified ^{1}H NMR spectrum recorded using the DMSO-d_6 extract of C_8 aromatics obtained from **sql-4,5-Zn** that was prior subjected to the equimolar binary liquid of PX/MX until saturated.

Since the peaks of MX and PX at 2.25 and 2.26 are combined and it is hard to get a good discrimination of these two compounds, so the peaks at 7.05 (four H atoms in benzene ring for PX) and 7.11, 7.13, 7.14 (one H atom in benzene ring for MX) were chosen to calculate the selectivity. The selectivity of PX/MX is 1.5.

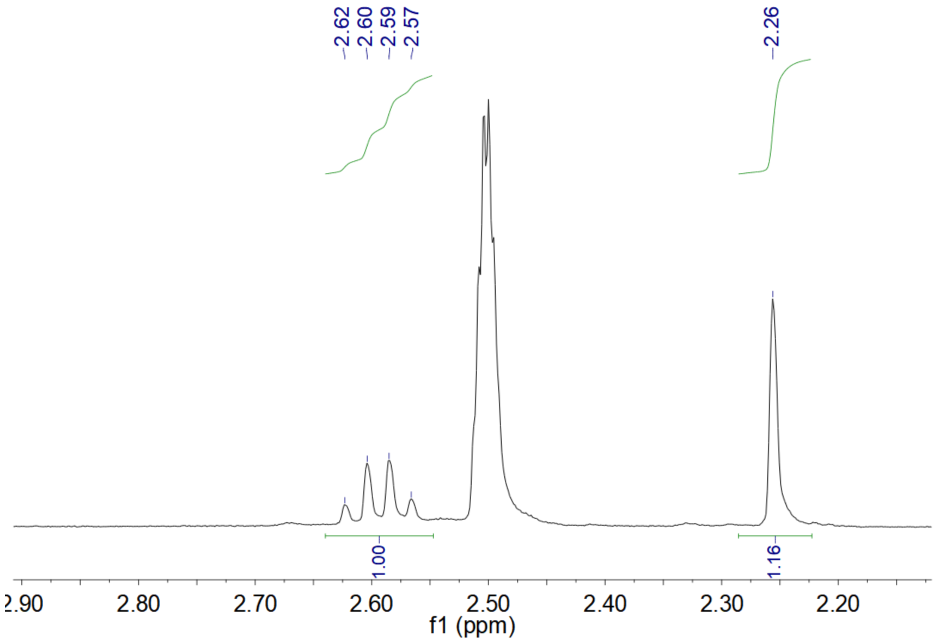

Figure S23. Magnified ^{1}H NMR spectrum recorded using the DMSO-d_6 extract of C_8 aromatics obtained from **sql-4,5-Zn** that was prior subjected to the equimolar binary liquid of EB/MX until saturated.

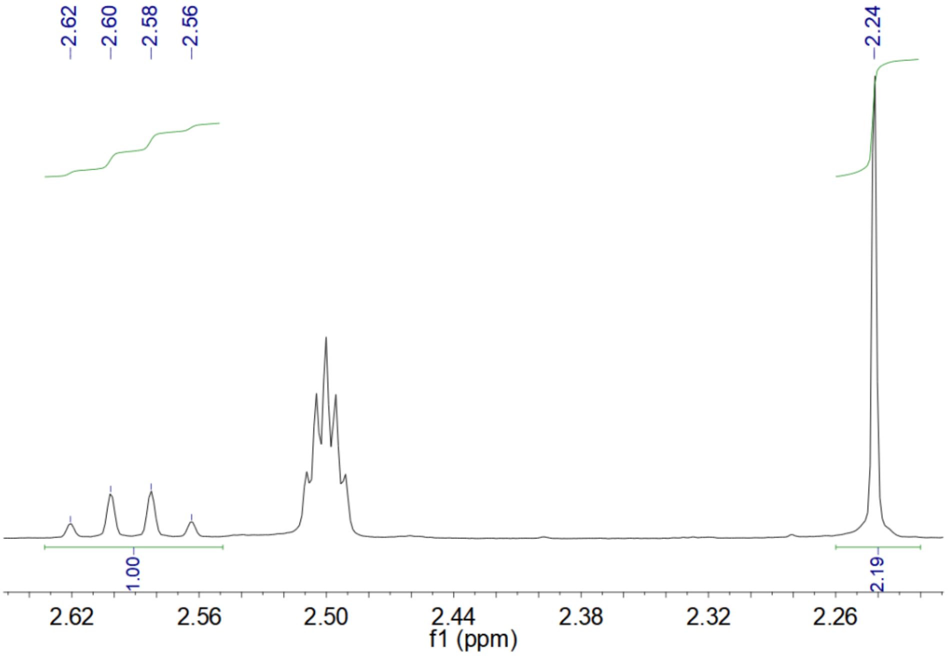

Figure S24. Magnified ^{1}H NMR spectrum recorded using the DMSO-d_6 extract of C_8 aromatics obtained from **sql-4,5-Zn** that was prior subjected to the equimolar binary liquid of EB/PX until saturated.

16.Langmuir fit

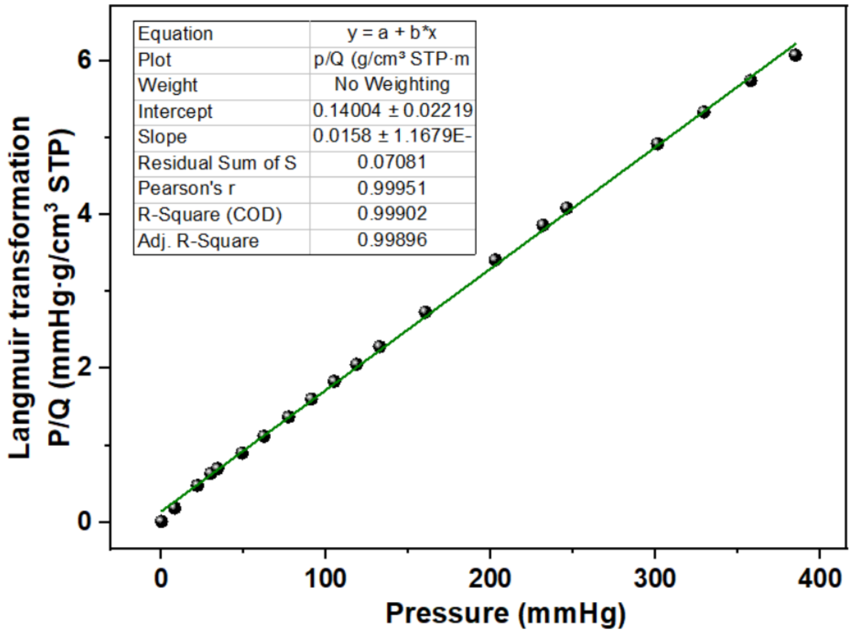

Figure S25. Langmuir fit corresponding to the first step from the 195 K CO_2 data for **sql-4,5-Zn-β**. Squares and line represent the experimental data and linear fitted data, respectively.

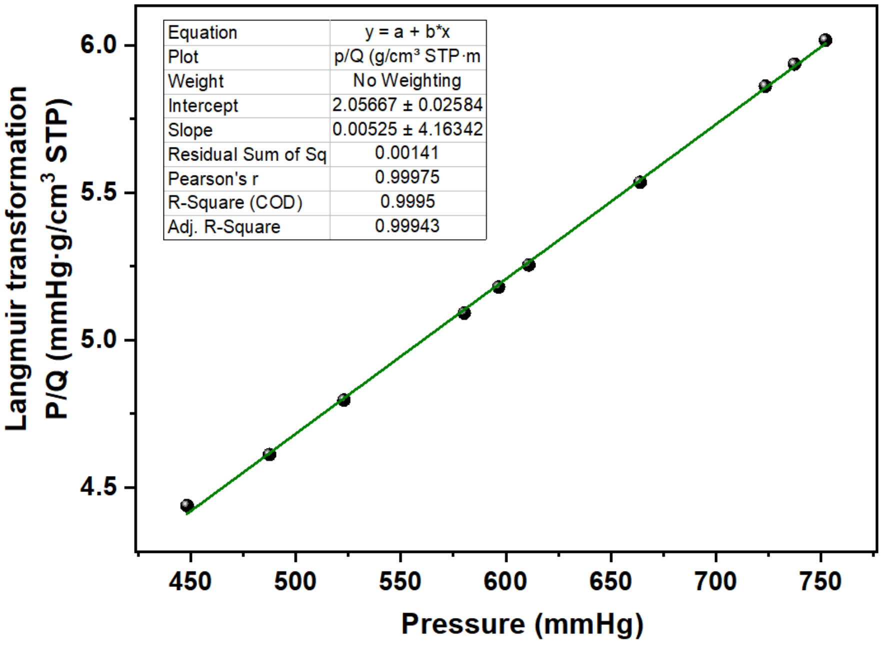

Figure S26. Langmuir fit corresponding to the second step from the 195 K CO_2 data for **sql-4,5-Zn-β**. Squares and line represent the experimental data and linear fitted data, respectively.

17.Stability Studies

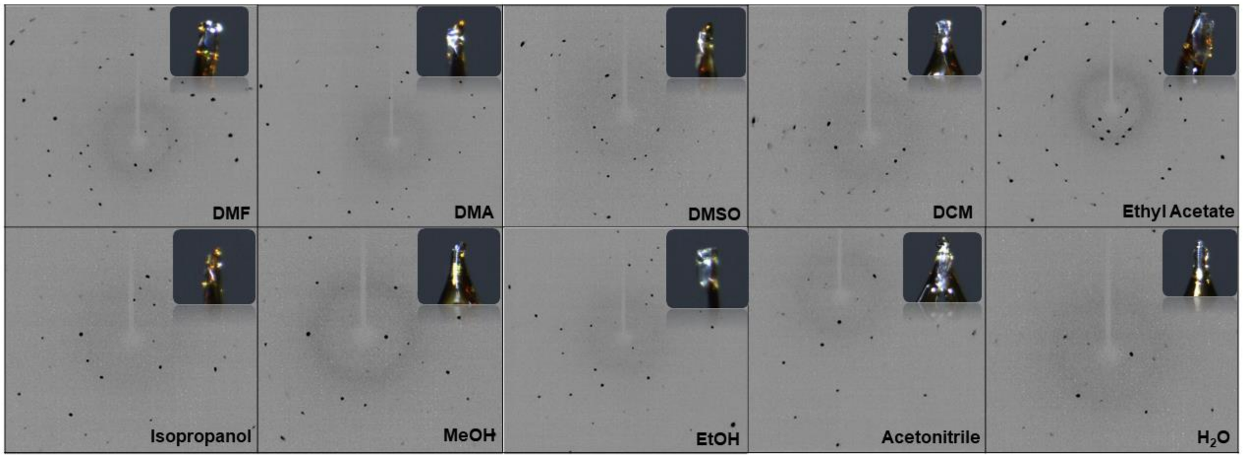

Figure S27. Diffraction image of the single crystals of **sql-4,5-Zn** after soaking in solvent for more than 24 hours. Single-crystal X-ray diffraction analysis reveals that retrieved crystal was still the original structure, fully confirmed the stability of **sql-4,5-Zn** in lots of solvents.

18.GC Spectra

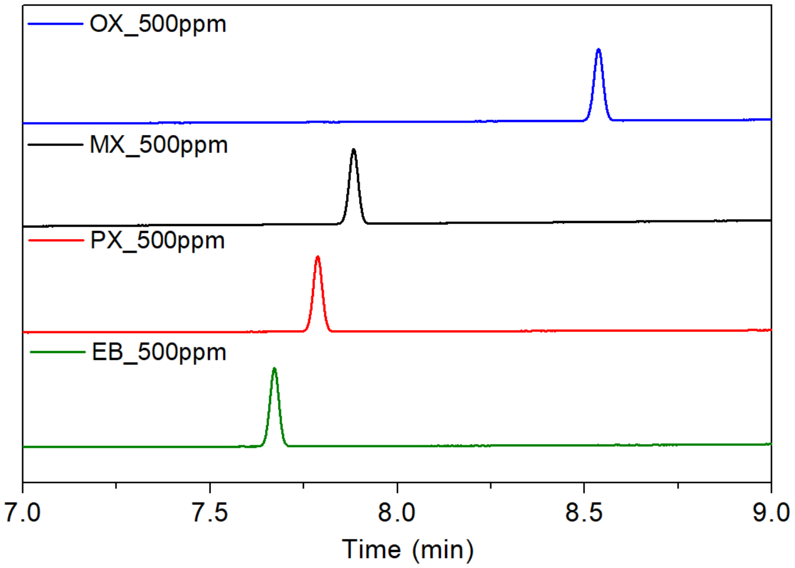

Figure S28. The spectra of standard solvents from GC tests.

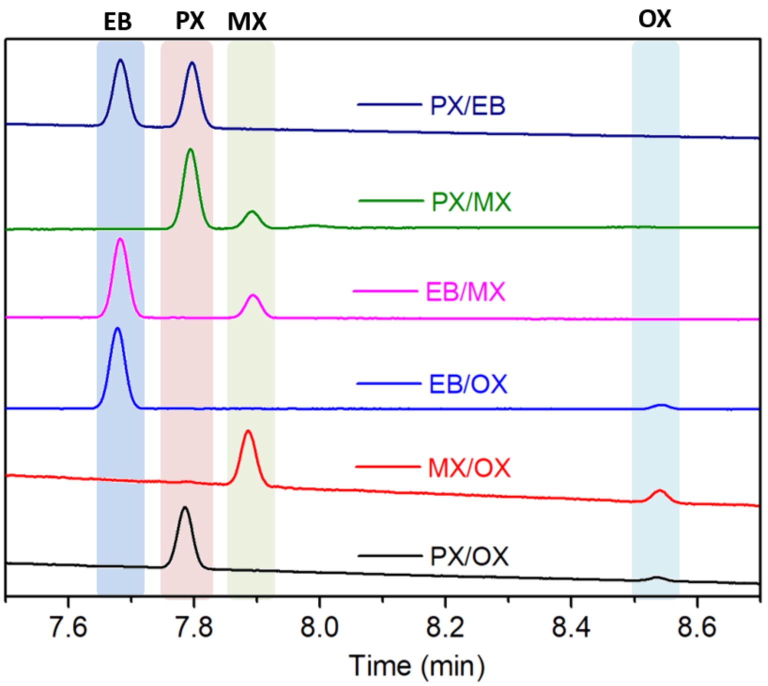

Figure S29. Gas chromatograms (GC) used to quantify the selectivity coefficients of **sql-4,5-Zn-β** after being soaked in the equimolar liquid binary mixtures.

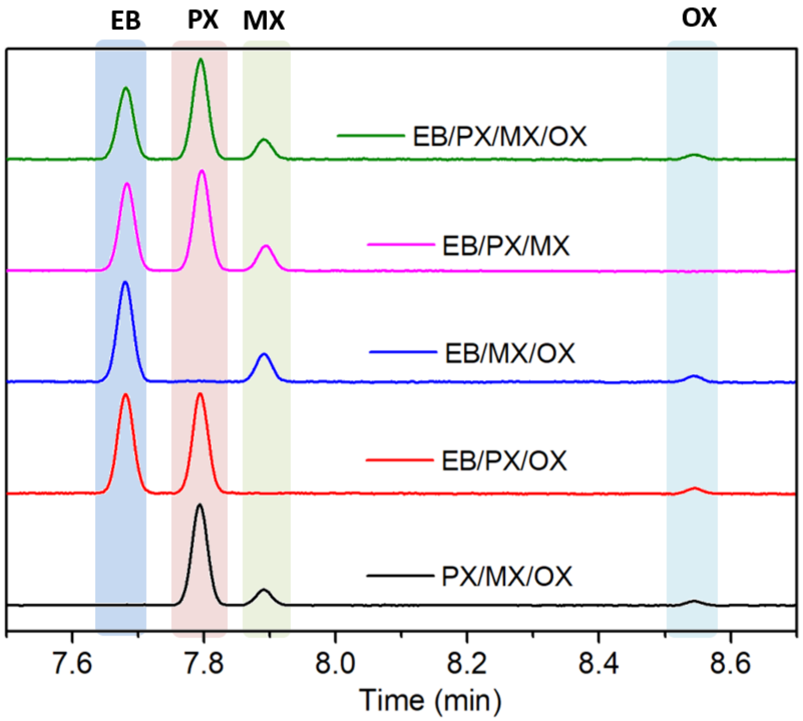

Figure S30. GC used to quantify the selectivity coefficients of **sql-4,5-Zn-β** after being soaked in the equimolar liquid ternary or quaternary mixtures.

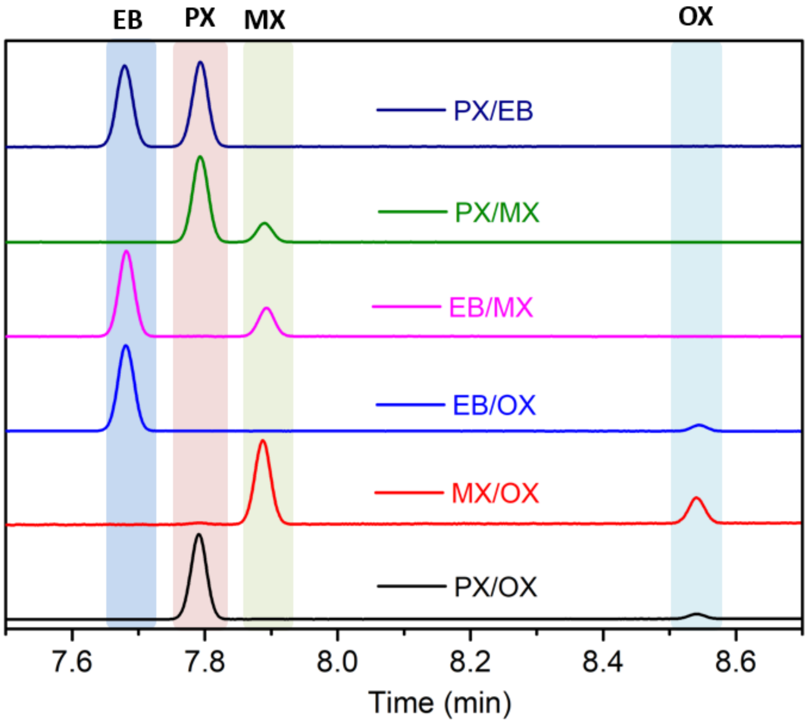

Figure S31. GC used to quantify the selectivity coefficients of **sql-4,5-Zn-β** that was prior subjected to the equimolar vapor binary mixtures until saturated.

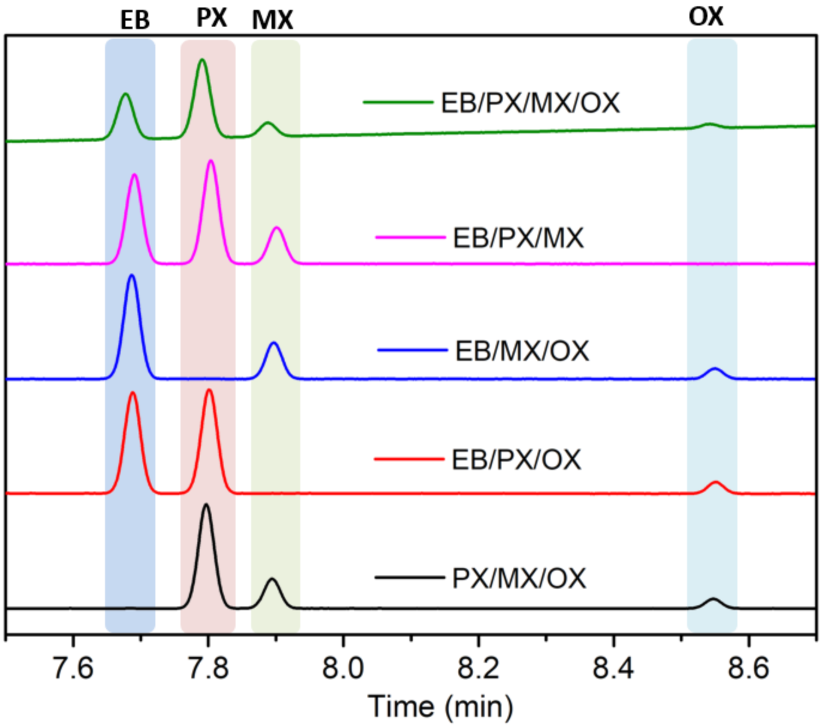

Figure S32. GC used to quantify the selectivity coefficients of **sql-4,5-Zn-β** that was prior subjected to the equimolar vapor ternary or quaternary mixtures until saturated.

19.Comparison of MOMs with separation of C_8 aromatics

Table S9. Selectivities of C_8 aromatics on some representative metal organic materials (MOMs).

Adsorbents	Dimention	Uptake (wt.%)				Selectivity			DOI
		OX	MX	PX	EB	PX/OX	EB/OX	MX/OX	
**MAF-89**	3D	15.9	18.6	21.2	NG	221[a]	NG	NG	10.1007/s11426-022-1304-1
**Zn(o-phen)(2,6-ndc)**	1D	35.4	43.8	41.1	0	64.6	NG	78.5	10.1002/anie.202106784
**$AgLClO_4$ (M)**	0D	0.2	0	8.5	NG	24.0	NG	3.93	10.1039/c9sc02621e
**H/ZSM-5**	3D	3.05	1.24	14.22	6.03	16.78[a]	NG	NG	10.1016/j.micromeso.2011.09.013
**MIL-120(Al)**	3D	0.8	0.8	5.3	2.1	11.5	NG	NG	10.1016/j.jiec.2022.10.021
**MAF-88**	3D	NG	NG	21.2	NG	10.7[a]	NG	NG	10.1007/s11426-022-1304-1
**Cu(CDC)**	3D	1	5.5	12	NG	10[a]	NG	3.0	10.1016/j.micromeso.2016.01.044
**$Ag_4(O_2CCF_3)_4$(phenazine)$_3$**	2D	NG	NG	NG	NG	9.13	NG	NG	10.1002/chem.201601870
**Li/ZSM-5**	3D	4.3	2.5	11	5.5	5.987[a]	NG	NG	10.1016/j.micromeso.2011.09.013
**[Ce(HTCPB)]**	3D	NG	12.7	11.7	NG	5.7[a]	NG	1.2	10.1002/anie.201307656
**Na/ZSM-5**	3D	3	2.5	9	5.5	5.415[a]	NG	NG	10.1016/j.micromeso.2011.09.013
**DUT-8(Cu)**	3D	8.5	8.5	19	8.5	5.4	NG	NG	10.1021/acsami.9b11343
**MOF-monoclinic**	3D	4.2	4.2	12.5	4.2	4.55[a]	NG	NG	10.1021/jp9063017
**K/ZSM-5**	3D	2	1.2	8.3	7.5	3.906[a]	NG	NG	10.1016/j.micromeso.2011.09.013
**ZIF-8**	3D	1.6	3.2	15.9	NG	3.9	NG	2.4	10.1016/j.micromeso.2013.01.012
**BaX nanosize**	3D	4.9	2.02	10.34	3.15	2.819[a]	NG	NG	10.1016/j.cherd.2013.10.008
**KaX nanosize**	3D	4.2	1.8	10.1	3.2	2.43[a]	NG	NG	10.1016/j.cjche.2014.11.005
**MIL-125(Ti)-NH_2**	3D	10	11	14.5	NG	2.2	NG	0.97	10.1021/ja207287h
**MIL-140B**	3D	12.7	12.7	12.7	12.7	1.8	NG	NG	10.1021/acs.jpcc.6b03349
**MOF-48**	3D	27.6	27.6	27.6	27.6	1.7	NG	NG	
**MCF-50**	1D	NG	NG	NG	NG	1.6[a]	NG	1.3	10.1038/srep11537
**sql-4,5-Zn**	**2D**	**20.7**	**15.9**	**5.0**	**3.3**	**8.0[b]**<br>**16.56[c]/13.69[d]**	**7.20[b]**<br>**13.65[c]/17.20[d**	**2.0[b]**<br>**3.43[c]/4.23[d]**	**This work**

Note: NG. refers to not given; [a] Measured by HPLC or GC; [b] Liquid phase binary mixture (equimolar) separation from ^{1}H NMR; [c] Vapor phase binary mixture (equimolar) separation from GC; [d] Liquid phase binary mixture (equimolar) separation from GC.

Table S10. Flexible or switching adsorbents for the separation of C_8 aromatics.

Adsorbents	Dimension	Year	DOI
**Ce(HTCPB)**	3D	2014	10.1002/anie.201307656
**MIL-53(Al/Cr/Ga)**	3D	2008	10.1021/ja802761z<br>10.1021/acs.jpcc.7b09105
**CAU-13**	3D	2014	10.1021/ic500288w
**MCF-50**	1D	2015	10.1038/srep11537
**Zn(o-phen)(2,6-ndc)**	1D	2021	10.1002/anie.202106784
**$Ni(NCS)_2(ppp)_4$**	0D	2012	10.1002/anie.201109084
**SAMM-3-Cu-OTf**	0D	2020	10.1039/C9CC09525J
**$Mn(dhbq)(H_2O)_2$**	0D	2022	10.1126/science.abj7659
**sql-1-Co-NCS**	2D	2019	10.1002/anie.201901198
**sql-1,3-Co-NCS**	2D	2020	10.1039/d0sc02123g
**sql-4,5-Zn**	**2D**	**-**	**This work**

Table S11. Some representative OX selective MOMs, arranged in descending order of year.

Adsorbent	Dimension	Uptake (wt%)				Selectivity			Year	Ref.
		OX	MX	PX	EB	OX/MX	OX/PX	OX/EB		
**ZUL-C3**	3D	36.1[a]	35.5[a]	35.5[a]	34.7[a]	7.92[a]	20.06[a]	7.51[a]	2022	10.1021/jacs.2c10595
**SIFSIX-1-Cu**	3D	13.0	11.2	7.9	NG	1.1	2.7	NG	2021	10.1002/chem.202100008
**MFM-300(In)**	3D	29.1	30	30.4	NG	1/2.9	1.6	NG	2020	10.1038/s41467-020-17640-4
**SAMM-3-Cu-OTf**	0D	42	0[b]	0[b]	0[b]	6.1	23.1	18.0	2020	10.1039/c9cc09525j
**ZU-61**	3D	3.2c	3.44[c]	3.37[c]	NG	2.9d	2.6d	NG	2020	10.1038/s41467-020-19209-7
**sql-1-Co-NCS**	2D	87.0	87.0	87.0	43.5	7.5	9.6	60.1	2019	10.1002/anie.201901198
**$Ni(NCS)_2(ppp)_4$**	0D	29	27	38	NG	34.2	40.5	NG	2012	10.1002/anie.201109084

**MIL-53(Cr)**	3D	42.4	27.6	42.4	25.4	2.8	3.7	4.9	2018	10.1021/acs.jpcc.7b09105
**MIL-53(Ga)**	3D	37.1	32.9	39.2	23.3	2.5	3.3	4.7	2018	10.1021/acs.jpcc.7b09105
**Co-MOF-74**	3D	38.2	36.0	35.0	35.0	2.5	3.9	1.21	2018	10.1021/jacs.7b13825
**CAU-13**	3D	17	15	14	NG	1.9	1.5	NG	2014	10.1021/ic500288w
**UiO-66**	3D	42.4	42.4	42.4	NG	1.8	2.4	NG	2012	10.1021/la3004118
**MOF-5**	3D	13	14.5	13	10	NG	NG	1.96	2010	10.1021/jp9063017
**MIL-53(Al)**	3D	46	26	43	17	2.7	3.5	10.9	2008	10.1021/ja802761z
**MIL-47(V)**	3D	35	28	37	16	2.0	1.4	10.9	2007	10.1002/anie.200700056

Note: NG. refers to not given; ^{a} 303 K; ^{b} no uptake; ^{c} 7.1 mbar, 333 K; ^{d} 398 K.

Table S12. Adsorbents showing stepwise removal of C_8 aromatics.

**Adsorbents**	**Dimension**	**Flexible or Switching**	**Number of Steps**				**SCXRD structures of IP phase**	**Year**	**Ref**
			OX	MX	PX	EB			
**$Ni(NCS)_2(ppp)_4$**	0D	Yes	2	2	2	NG	No	2012	10.1002/anie.201109084
**sql-1-Co-NCS**	2D	Yes	3	3	4	2	No	2019	10.1002/anie.201901198
**sql-4,5-Zn**	**2D**	**Yes**	**2**	**2**	**2**	**2**	**Yes**	**-**	**This work**

Note: NG. refers to not given.

20. Interactions between Framework and C_8 Aromatics.

Table S13. A summary of interaction between C_8 aromatics and framework.

**C_8 aromatics**	**Located**	**Type**	**Distance (Å)**
**OX**	In square cavities	π···π	4.313
		C-H···π[a]	3.858, 3.715, 3.386, 4.182
		π···O	4.077
	In interlayer/intralayer spaces	π···π	No
		C-H···π	3.896, 3.650, 4.068
**MX**	In square cavities	π···π	4.654, 4.577

		C-H···π	3.728, 3.840, 4.020
		π···O	No
	In interlayer/intralayer spaces	π···π	No
		C-H···π	3.970
**PX**	In square cavities	π···π	4.684, 4.595
		C-H···π	4.122, 4.008
		π···O	4.202, 4.237
	In interlayer/intralayer spaces	π···π	4.559
		C-H···π	3.648, 4.092, 4.185, 3.560
**EB**	In square cavities	π···π	4.043
		C-H···π	4.139, 3.977, 3.726, 3.915, 4.138
		π···O	3.986
	In interlayer/intralayer spaces	π···π	No
		C-H···π	3.977, 4.102, 3.977

[a] The distance of C-H···*π* is measured from C atom to the aromatic ring centroid

21. Separation results from GC and ^{1}H NMR

Table S14. Separation performance of binary, ternary and quaternary C8 aromatics (liquid phase, equimolar) from GC Result.

	**C_8 aromatics**	**Ratio**	**Selectivity**
Binary	PX/OX	13.69:1	13.69
	EB/OX	16.92:1	16.92
	MX/OX	4.23:1	4.23
	EB/MX	3.36:1	3.36
	PX/MX	4.50:1	4.50
	EB/PX	1.03:1	1.03
Ternary	PX/MX/OX	19.01:3.08:1	9.31
	EB/PX/OX	16.23:16.12:1	1.90
	EB/MX/OX	12.49:3.65:1	5.37
	EB/PX/MX	3.93/3.83/1	1.63
Quaternary	PX/EB/MX/OX	18.53:13.67:3.96:1	2.99

Table S15. Separation performance of binary C8 aromatics (vapor phase, equimolar) from GC Result at 303K.

	C_8 aromatics	Ratio	Selectivity
Binary	PX/OX	16.56:1	16.56
	EB/OX	13.65:1	13.65
	MX/OX	3.43:1	3.43
	EB/MX	2.90:1	2.90
	PX/MX	4.51:1	4.51
	EB/PX	1.02:1	1.02
Ternary	PX/MX/OX	10.34:2.97:1	5.21
	EB/PX/OX	8.300:8.626:1	1.27
	EB/MX/OX	9.624:3.372:1	4.40
	EB/PX/MX	2.386:2.763:1	1.72
Quaternary	PX/EB/MX/OX	17.14:10.11:3.11:1	3.62

Table S16. Separation performance of binary C8 aromatics (liquid phase) from ^{1}H NMR.

	C_8 aromatics	Ratio	Selectivity
Binary	PX/OX	8.0:1	8.0
	EB/OX	7.20:1	7.20
	MX/OX	2.0:1	2.0
	EB/MX	2.59:1	2.59
	PX/MX	1.50:1	1.50
	EB/PX	1.37:1	1.37

22. Coefficients for Antoine equation.

Table S17. The values of A, B and C for C8 aromatics from the Antoine equation.

C8 aromatics	A	B	C
OX	6.99891	1474.679	213.686
MX	7.00908	1462.266	215.105
PX	6.99052	1453.43000	215.307
EB	6.95719	1424.255	213.206

23.References

1. Bondi, A. van der Waals Volumes and Radii. *The Journal of Physical Chemistry*. **1964**, *68*, 441-451.
2. Krause, L.; Herbst-Irmer, R.; Sheldrick, G. M.; Stalke, D. Comparison of silver and molybdenum microfocus X-ray sources for single-crystal structure determination. *Journal of Applied Crystallography*. **2015**, *48*, 3-10.
3. Gannon, R. E.; Krukonis, V. J.; Schoenberg, T. Conversion of Coal to Acetylene in Arc-Heated Hydrogen. *Product R&D.* **1970**, *9*, 343-347.
4. Sheldrick, G. SHELXT - Integrated space-group and crystal-structure determination. *Acta Crystallographica Section A.* **2015**, *71*, 3-8.
5. Sheldrick, G. Crystal structure refinement with SHELXL. *Acta Crystallographica Section C.* **2015**, *71*, 3-8.
6. Dolomanov, O. V.; Bourhis, L. J.; Gildea, R. J.; Howard, J. A. K.; Puschmann, H. OLEX2: a complete structure solution, refinement and analysis program. *Journal of Applied Crystallography*. **2009**, *42*, 339-341.
7. Wang, S. Q.; Mukherjee, S.; Patyk-Kazmierczak, E.; Darwish, S.; Bajpai, A.; Yang, Q. Y.; Zaworotko, M. J. Highly Selective, High-Capacity Separation of o-Xylene from C_8 Aromatics by a Switching Adsorbent Layered Material. *Angew. Chem. Int. Ed.* **2019,** *58*, 6630-6634.
8. V. A. Blatov, A. P. Shevchenko, D. M. Proserpio, *Crystal Growth & Design* 2014, **14**, 3576-3586.
9. C. R. Groom, I. J. Bruno, M. P. Lightfoot, S. C. Ward, *Acta Cryst. B* 2016, **72**, 171-179.
10. Allen, F. H.; Motherwell, W. D. Applications of the Cambridge Structural Database in organic chemistry and crystal chemistry. *Acta Crystallogr B.* **2002,** *58*, 407-22.
11. Moghadam, P. Z.; Li, A.; Wiggin, S. B.; Tao, A.; Maloney, A. G. P.; Wood, P. A.; Ward, S. C.; Fairen-Jimenez, D. Development of a Cambridge Structural Database Subset: A Collection of Metal-Organic Frameworks for Past, Present, and Future. *Chem. Mater.* **2017,** *29*, 2618-2625.
12. Allen, F. H.; Taylor, R. Research applications of the Cambridge Structural Database (CSD). *Chem. Soc. Rev.* **2004,** *33*, 463-75.
